# Recent Advances in Transparent Electrodes and Their Multimodal Sensing Applications

**DOI:** 10.1002/advs.202405099

**Published:** 2024-08-09

**Authors:** Majed Althumayri, Ritu Das, Ramu Banavath, Levent Beker, Alin M. Achim, Hatice Ceylan Koydemir

**Affiliations:** ^1^ Department of Biomedical Engineering Texas A&M University College Station TX 77843 USA; ^2^ Center for Remote Health Technologies and Systems Texas A&M Engineering Experiment Station College Station TX 77843 USA; ^3^ Department of Mechanical Engineering Koç University Sariyer Istanbul 34450 Turkey; ^4^ School of Computer Science University of Bristol Bristol BS8 1QU UK

**Keywords:** multimodal sensing, optoelectrical sensing, optoacoustic sensing, optomechanical sensing, quantum sensing, transparent materials

## Abstract

This review examines the recent advancements in transparent electrodes and their crucial role in multimodal sensing technologies. Transparent electrodes, notable for their optical transparency and electrical conductivity, are revolutionizing sensors by enabling the simultaneous detection of diverse physical, chemical, and biological signals. Materials like graphene, carbon nanotubes, and conductive polymers, which offer a balance between optical transparency, electrical conductivity, and mechanical flexibility, are at the forefront of this development. These electrodes are integral in various applications, from healthcare to solar cell technologies, enhancing sensor performance in complex environments. The paper addresses challenges in applying these electrodes, such as the need for mechanical flexibility, high optoelectronic performance, and biocompatibility. It explores new materials and innovative techniques to overcome these hurdles, aiming to broaden the capabilities of multimodal sensing devices. The review provides a comparative analysis of different transparent electrode materials, discussing their applications and the ongoing development of novel electrode systems for multimodal sensing. This exploration offers insights into future advancements in transparent electrodes, highlighting their transformative potential in bioelectronics and multimodal sensing technologies.

## Graphical abstract

This review evaluates recent advancements in transparent electrodes, emphasizing their important role in multimodal sensing technologies. Highlighting materials such as graphene, carbon nanotubes, and conductive polymers, it explores their applications across various sectors, including healthcare and environmental monitoring. The paper discusses material properties, challenges, and innovative solutions, providing insights into future developments in bioelectronics and sensor technology.

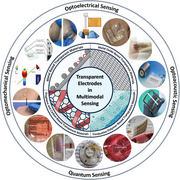

## 1 Introduction

Transparent electrodes, which are materials that allow light to pass through while also conducting electricity, have gained importance recently in bioelectronic devices due to their exceptional optoelectrical properties, such as high transmittance and low electrical resistance.^[^ ^1^, ^2^, ^3^, ^4^, ^5^, ^6^ ^]^ Alongside these properties, transparent electrodes possess mechanical robustness and flexibility, allowing them to endure bending and stretching, which is critical for maintaining functionality in dynamic applications, making them excellent substrates for multimodal sensing applications.^[^ ^7^, ^8^ ^]^ Moreover, transparent electrodes act as a crucial interface that facilitates the interaction between light and electronic circuits. This role is critical in devices ranging from interactive displays to photovoltaic cells, where high transparency is necessary to allow for the passage of light, and low electrical resistance is crucial for the efficient transmission of electrical signals.^[^ ^9^ ^]^ As a result, research has turned toward materials such as graphene, carbon nanotubes, and conductive polymers, which are known for their potential to offer a balance of optical transparency, electrical conductivity, and mechanical flexibility, essential for the next generation of flexible and wearable electronic devices.^[^ ^10^ ^]^

Multimodal sensing technologies represent a cutting‐edge frontier enabling the simultaneous detection and analysis of various physical, chemical, and biological signals. These technologies are important in enhancing the sensitivity and specificity of sensors, as they can gather multifaceted information from reactions occurring on the sensor's surface.^[^ ^11^, ^12^ ^]^ The integration of transparent electrodes in these applications is crucial, as they facilitate the simultaneous detection of multiple target analytes on the same substrate, utilizing different sensing mechanisms.^[^ ^13^ ^]^ This capability not only broadens the scope of potential applications but also significantly improves the performance of sensors in complex environments.^[^ ^14^ ^]^

In healthcare and biosensing applications, transparent electrodes are integral not only to the development of smart wearable and implantable optoelectronic devices but also to portable sensing platforms such as tissue cell culture imaging and microfluidic applications.^[^ ^15^, ^16^, ^17^, ^18^ ^]^ Additionally, these materials are increasingly being recognized for their significance in advanced contact lens technologies, where they provide new features like ongoing health monitoring.^[^ ^19^, ^20^, ^21^ ^]^ The role of these materials is to facilitate the transfer of both electrical and mechanical signals while maintaining transparency for optical measurements.^[^ ^13^, ^22^ ^]^

These electrodes have the unique capability of allowing multimodal assessments with electrophysiological, electrochemical, and optical measures for detailed monitoring of physiological and biological functions.^[^ ^13^, ^23^, ^24^ ^]^ Nevertheless, there are a number of difficulties when using these electrodes for flexible sensing applications. First, wearable or implantable sensors are often attached to human skin or organs, which are dynamic and curved surfaces. Therefore, maintaining conformal and continuous contact between the sensor and the human body is essential in order to obtain accurate bio‐signals.^[^ ^25^, ^26^ ^]^ This requires a significant degree of mechanical flexibility to accommodate the diverse geometries of biological surfaces. For instance, emerging materials, e.g., carbon nanotubes (CNTs) and graphene, are being utilized due to their advantages such as exceptional flexibility, strength, and conductivity.^[^ ^27^, ^28^ ^]^ Additionally, silver nanowires (AgNWs) are emerging as a preferred material because of their high electrical conductivity and ability to form a percolation network that remains conductive even under deformation.^[^ ^29^ ^]^ Flexible polymers such as poly(3,4‐ethylenedioxythiophene) polystyrene sulfonate (PEDOT) are also being explored for their biocompatibility and mechanical properties.^[^ ^30^ ^]^ These materials are often combined with innovative designs such as kirigami engineered mesh structures,^[^ ^31^ ^]^ wavy configurations, and stretchable interconnects that allow the electrodes to stretch, bend, and twist while maintaining their functionality.^[^ ^32^, ^33^ ^]^

Additionally, achieving high optoelectronic performance, marked by substantial visible transparency and robust electrical conductivity, is crucial for effective signal transmission and aesthetic purposes.^[^ ^34^ ^]^ This is particularly relevant in ultraviolet (UV) optoelectronic devices, where gallium(III) oxide (Ga_2_O_3_)/silver (Ag)/Ga_2_O_3_ laminated films show promise, and in flexible electronics, where silver nanowires are becoming a preferred choice over traditional materials, offering cost‐effectiveness and enhanced flexibility.^[^ ^35^, ^36^ ^]^ The low cost and simple fabrication of transparent flexible electrode materials are other challenges to be considered for the mass production and easy prototyping of multimodal sensing systems.^[^ ^37^, ^38^, ^39^ ^]^ Addressing these challenges requires a closer look at the materials currently employed in transparent electrodes, as well as the exploration of new alternatives that can meet the demanding requirements of multimodal sensing devices.

**Figure 1:**
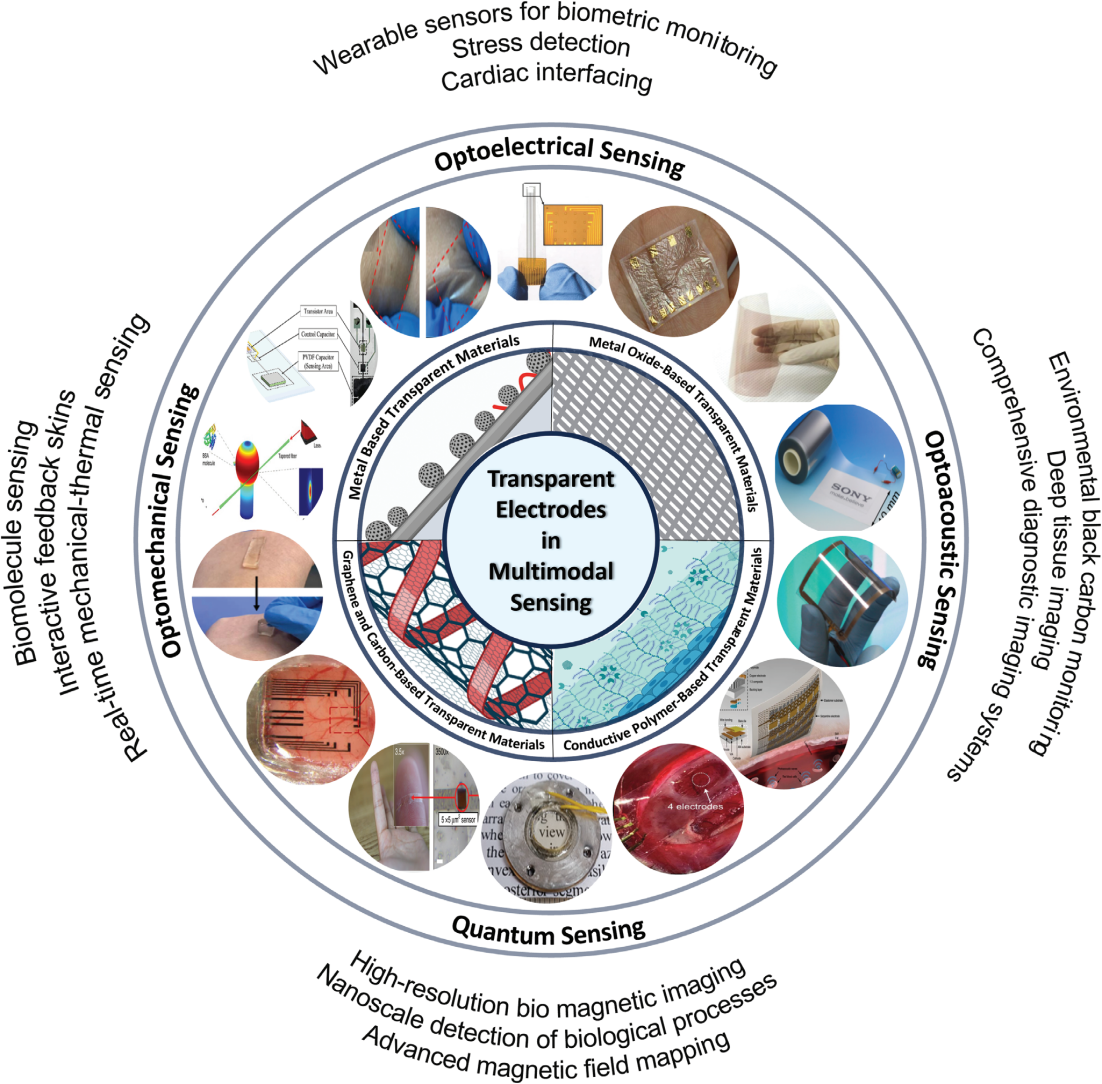
Overview of transparent electrodes, including metal‐oxide‐based materials, metal‐based materials, conductive polymer‐based materials, and graphene and carbon‐based materials in their multimodal sensing applications.

This review aims to provide a thorough understanding of the significance of transparent electrode materials in the context of multimodal electronic systems. The review is organized to start by talking about how important multimodal sensing applications are and how important transparent electrode materials are. Next, we will discuss the developments on new transparent electrode systems based on smart multimodal sensing applications (**Figure** **1**). The current state and advancements in transparent electrode materials will be covered, with an emphasis on how multimodal sensing might benefit from them. Finally, we will discuss difficulties and potential prospects facing this field going forward and provide a thorough comparison of various materials and technologies. This review is intended for researchers and professionals in the fields of materials science, bioelectronics, and sensor technology, focusing particularly on those interested in the latest developments in transparent electrodes and their applications in advanced sensing systems.

## 2 Overview of Transparent Electrodes

Transparent electrodes (TEs) are indispensable in optoelectronic devices,^[^ ^40^, ^41^ ^]^ such as solar cells,^[^ ^42^ ^]^ touch panels,^[^ ^43^ ^]^ smart windows,^[^ ^44^ ^]^ and wearable electronics.^[^ ^45^ ^]^ While these applications pertain to optoelectronic devices, the advancements in transparent electrode materials are directly relevant to the development of biosensors. The same properties that enhance performance in optoelectronic devices—such as high transparency, conductivity, and flexibility—are crucial for creating more effective biosensors. These properties enable the simultaneous detection and measurement of biological signals, improving the sensitivity and specificity of biosensors. Therefore, the progress in developing transparent electrodes for optoelectronic applications also drives innovations in biosensor technology, underscoring the interdisciplinary nature of advancements in this field.

The quest to develop efficient and versatile transparent electrode materials has led to exploring various materials, each offering unique properties and potential applications. This section delves into the materials that have emerged as potential candidates for transparent electrodes, examining their properties, advantages, and challenges. Transparent electrodes can be broadly divided into a number of groups based on the materials used: metal oxide‐based electrodes, metal‐based electrodes, conductive polymer‐based electrodes, and carbon‐based electrodes. Each category offers unique advantages and challenges, such as the high conductivity and transparency of metal oxides like indium tin oxide (ITO), the flexibility and conductivity of metal nanowires, the processability of conductive polymers like PEDOT, and the balance of properties provided by carbon‐based materials like graphene and carbon nanotubes. These classifications guide the development of next‐generation transparent electrodes for various advanced applications.

### 2.1 Metal Oxide‐Based Transparent Electrodes Materials

Metal oxide‐based materials, compounds formed of metallic elements combined with oxygen, have risen to prominence in developing these electrodes.^[^ ^40^, ^41^ ^]^ Their unique optoelectronic properties, encompassing a spectrum of electrical conductivities and engineered optical transparency in the visible range, make them particularly apt for these applications.^[^ ^46^, ^47^ ^]^ Table S1 (Supporting Information) lists emerging metal oxide‐based transparent materials, their properties, and applications.

**Figure 2:**
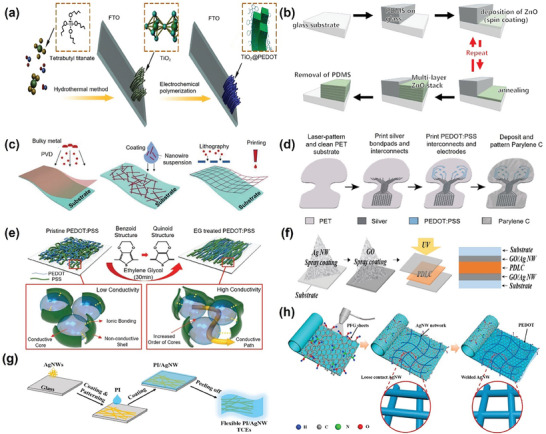
Fabrication steps to create a) titanium dioxide (TiO_2_)/poly(3,4‐ethylenedioxythiophene) (PEDOT) layer used for energy storage devices. Reprinted with permission from ref. [61], b) multi‐layer ZnO stack for optoelectronic device fabrication^[^ ^51^ ^]^ © 2021 Wiley‐VCH GmbH, c) metal‐based electrode structures on a substrate^[^ ^68^ ^]^ © 2021 Wiley‐VCH GmbH, d) electrode arrays on planar polyethylene terephthalate substrates^[^ ^116^ ^]^ © 2022 The Authors. Advanced Healthcare Materials published by Wiley‐VCH GmbH, e) ethylene glycol‐induced morphological changes in PEDOT: PSS and its molecular‐level impact on conductivity^[^ ^115^ ^]^ © 2021 The Authors. Advanced Functional Materials published by Wiley‐VCH GmbH, f) silver nanowire–graphene oxide hybrid electrodes in smart windows using polymer‐dispersed liquid crystals,^[^ ^101^ ^]^ g) polyimide/silver nanowires transparent electrodes,^[^ ^149^ ^]^ and h) the structure of graphene and AgNWs composite for transparent electrodes Reprinted from ref. [283]. Copyright 2018 with permission from Elsevier.

ITO is the most prevalent conductive oxide. Its widespread adoption is attributed to its superior conductivity and transparency. However, challenges such as the scarcity of indium, the brittle nature of ITO films, and high processing costs have prompted the exploration of alternatives.^[^ ^48^, ^49^ ^]^ Zinc oxide (ZnO), aluminum zinc oxide (AZO), gallium zinc oxide (GZO), and fluorine‐doped tin oxide (FTO) are now under the spotlight as these alternatives present unique properties and offer solutions to ITO's limitations.^[^ ^50^ ^]^ For instance, the challenge of achieving high conductivity in transparent electrodes has been addressed by using UV‐treated sol–gel‐grown ZnO films. This treatment significantly enhances the films' conductivity, as shown in **Figure** **2**a, where the conductivity of the sol–gel‐grown ZnO film is plotted as a function of UV treatment time.^[^ ^51^ ^]^

Research has delved into understanding the nuances of these materials where a study on the optimal thickness of ITO for near‐ultraviolet (NUV) light emitting diodes (LEDs) revealed that a 110 nm ITO film, annealed at 550 °C for a minute, showcased the highest figure of merit.^[^ ^52^ ^]^ Another innovation combined AZO with silver nanowires, emphasizing its potential for diverse sensing platforms.^[^ ^53^ ^]^ Further studies have investigated the influence of surface morphology on the optical properties of doped and undoped tin (IV) oxide (SnO_2_) thin films.^[^ ^54^ ^]^

**Figure 3:**
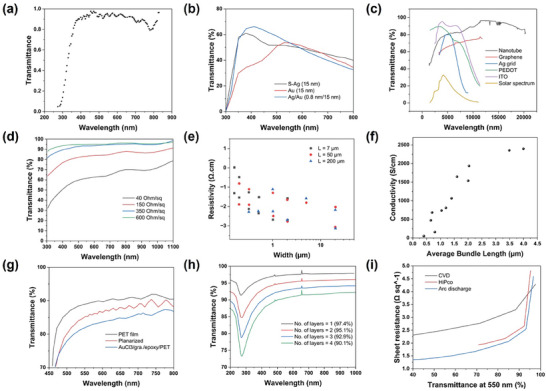
a) Optical transmittance of Indium tin oxide films, showcasing optical performance. Reprinted with permission from ref. [55], b) Transmittance of silver and gold‐based metal films at different wavelengths^[^ ^75^ ^]^ © 2017 WILEY‐VCH Verlag GmbH & Co. KGaA, Weinheim, c) Transmittance comparison of carbon‐based materials with other transparent conductive materials over a wide wavelength range (The *y*‐axis of the solar spectrum is not transmittance but irradiance in W m^−2^ nm^−1^). Reprinted with permission from ref. [284], d) Transmittance as a function of wavelength across the range of 300–1100 nm, varying with changes in carbon nanotube density and conductivity. Carbon nanotube density refers to the quantity of carbon nanotubes per unit area of the film, with the inset showcasing a carbon nanotube film on a flexible polyethylene terephthalate substrate Reprinted with permission from ref. [168]. Copyright 2006 American Chemical Society, e) a log‐log plot detailing the relationship between resistivity and the channel width of carbon nanotube films. The films exhibit various thicknesses, specifically 15 and 35 nm, along with different lengths (200, 50, and 7 µm) Reprinted from ref. [174], with the permission of AIP Publishing, f) the conductivity of the carbon nanotube film at different lengths of carbon nanotube bundles Reprinted from ref. [173], with the permission of AIP Publishing, g) Transparent properties of fabricated PET film Reprinted from ref. [188], with the permission of AIP Publishing, h) The transparency of roll‐to‐roll layer‐by‐layer graphene‐based transparent electrodes improved using the doping process. Reprinted with permission from ref. [187], and i) Impact of carbon nanotube synthesis methods, including chemical vapor deposition (CVD), high‐pressure carbon monoxide (HiPco), and arc discharge, on the properties of transparent conductive films. Reprinted with permission from ref. [285]. Copyright 2016 American Chemical Society.

**Figure 4:**
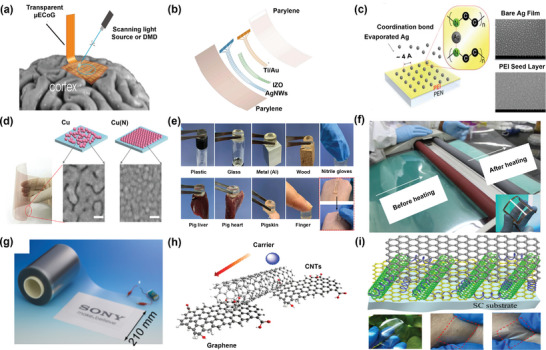
a) Transparent electrode application in brain‐machine interfacing. Reprinted with permission from ref. [55], b) Layered fabrication process of an electrocorticography electrode array. Reprinted with permission from ref. [59]. Copyright 2021 American Chemical Society, c) Seed layer strategy for the uniform metal film; conceptual diagram for the growth mechanism of the silver film with the polyethyleneimine (PEI) nucleation inducer. Sides: SEM images of the bare‐silver and PEI–silver electrodes,^[^ ^92^ ^]^ d) Doping strategy for the uniform metal film; Copper‐based flexible transparent electrodes, Top: diagrams representing the different growth modes of pure copper and Nitrogen‐doped copper films. Bottom: SEM images of the 6.5 nm‐thick copper and Nitrogen‐doped copper films on polyethylene terephthalate substrate (Scale bars, 20 nm)^[^ ^95^ ^]^ © 2016 WILEY‐VCH Verlag GmbH & Co. KGaA, Weinheim, e) Images demonstrating a hydrogel based transparent electrode adhesion to various substrates and tissues Reprinted with permission from ref. [151]. Copyright 2023 American Chemical Society, f) Roll‐to‐roll transfer of graphene films from a thermal release tape to a polyethylene terephthalate film at 120 °C and inset shows the ultrathin graphene film on polyethylene terephthalate. Reprinted with permission from ref. [187], g) Photograph of the graphene/epoxy/ polyethylene terephthalate transparent film roll fabricated by SONY Reprinted from ref. [188], with the permission of AIP Publishing. h) The structure of CNT and graphene‐based composite for transparent electrodes Reprinted with permission from ref. [200]. Copyright 2009 American Chemical Society. i) The structure of graphene and conductive polymer (PEDOT:PSS) composite‐based transparent electrodes and the below images show the fabricated graphene and conductive polymer composite‐based flexible and stretchable transparent electrodes Reprinted with permission from ref. [202]. Copyright 2018 American Chemical Society.

ITO's high transmittance and low autofluorescence, providing up to 90% transparency across the visible spectrum, have made it a staple in various domains, from LEDs and displays to solar cells and microelectrodes.^[^ ^55^ ^]^ The detailed optical properties of ITO are shown in (**Figure** **3**a). Its application in fabricating transparent micro‐electrocorticography (µECoG) arrays used in brain‐machine interfaces is particularly noteworthy, as shown in (**Figure** **4**a), which depicts the transparent µECoG array on the brain surface.^[^ ^55^ ^]^ However, the scarcity and cost of ITO present sustainability challenges.^[^ ^55^ ^]^ Indium is a relatively rare element, and its extraction and processing are energy‐intensive and environmentally detrimental. Hybrid materials combining ITO with conductive polymers or carbon‐based materials like graphene and CNTs are being investigated to reduce indium content while maintaining performance.^[^ ^56^ ^]^ Recycling and reclaiming ITO from end‐of‐life electronic devices is another strategy to mitigate scarcity and reduce environmental impact.^[^ ^57^ ^]^ While enhancing its properties, augmenting ITO with elements like gold introduces potential issues like oxidation that could impact long‐term stability.^[^ ^58^ ^]^ Integrating ITO with materials like AgNWs and indium‐zinc oxide (IZO) offers a stable, flexible electrode suitable for physiological conditions, as shown in (Figure 4b). However, the biocompatibility of AgNWs over extended periods remains a concern.^[^ ^59^ ^]^ On the other hand, ZnO crystal electrodes, vital for optogenetic studies, have challenges, such as instability under certain conditions.^[^ ^60^ ^]^

Innovative approaches, for example, using 1D titanium dioxide (TiO_2_) nanorod arrays as templates for PEDOT nanocomposites have been introduced for electrochromic energy storage devices.^[^ ^61^ ^]^ These devices outperform pure PEDOT films in several metrics, though their intricate fabrication process might limit their widespread use.^[^ ^61^ ^]^ ZnO stack can be grown using the sequential deposition strategy, a crucial step in this innovative approach, is detailed in (Figure 2b), and can be patterned using polydimethylsiloxane (PDMS), which is later used for constructing optoelectronic devices.^[^ ^51^ ^]^

While significant strides have been made in the domain of metal oxide‐based transparent electrodes, challenges persist. Each material, with its inherent advantages and limitations, offers insights into potential future research directions, aiming to push the envelope in electrochemical applications. Additionally, the biocompatibility of these materials can vary. For instance, ITO is generally considered biocompatible, but concerns about indium leaching have prompted research into alternatives like ZnO and FTO. ZnO, in particular, has shown promise due to its antibacterial properties and low toxicity in various biomedical applications.^[^ ^62^ ^]^ However, the long‐term stability and potential for degradation under physiological conditions remain areas of active research.

### 2.2 Metal‐Based Transparent Electrode Materials

Metal‐based TEs have been advanced in the last few decades on flexible electronics, and they can now be seen as promising candidates for flexible optoelectronic devices.^[^ ^63^, ^64^ ^]^ Metals possess the highest conductivity compared to other materials, allowing them to make flexible transparent electrodes by changing their structures at the nanometric level and creating ultrathin films and porous structures.^[^ ^65^, ^66^ ^]^ Figure 3b indicates the high transmittance and conductivity of metal‐based thin films. Metal‐based materials are the most promising ones for flexible transparent electrodes (FTEs) due to their high conductivity and tunable transmittance and flexibility.^[^ ^67^ ^]^ Different metals like silver (Ag), gold (Au), copper (Cu), and aluminum (Al) have been used for the fabrication of ultrathin metal films with thicknesses between 10 and 20 nm. Among these materials, Ag and Cu are most frequently used as FTEs materials for optoelectronic applications.^[^ ^68^ ^]^ Different engineered metal microstructures, for example, ultrathin film, nanowires, mesh, and composite structures, have also been used as flexible transparent electrodes, as shown in (Figure 2c).^[^ ^63^ ^]^ Table S2 (Supporting Information) presents a summary list of metal‐based transparent materials used in literature.

In general, metal films possess good mechanical robustness and low sheet resistance due to their inherent properties similar to bulk metal. However, the fabrication of ultrathin transparent metal films by physical vapor deposition (PVD) requires optimization. PVDs follow the Volmer–Weber growth. This mechanism involves the agglomeration of metal atoms into isolated islands instead of forming a continuous film layer.^[^ ^69^ ^]^ For continuous thin films, percolation thresholds are typically between 10 and 20 nm.^[^ ^70^ ^]^ Before the percolation threshold is reached, the metal layer exhibits very high sheet resistance. Although further metal deposition will significantly reduce the sheet resistance, there will also be a dramatic fall in optical transmittance. To achieve high‐performing, flexible, and transparent electrodes made of ultrathin metal films, it is important to improve the wettability of metals on the substrate and reduce the percolation threshold.^[^ ^64^, ^71^ ^]^

Two main strategies, seeding a layer and doping, have been tried to improve metal wettability during PVD on substrates to get homogeneous, smooth, thin films (Figure 4c,d). In the seed layer approach, a thin seed layer is deposited on the substrate surface before the PVD of metal films. These seed layers serve as concentrated nucleation points for metal deposition, facilitating the formation of metal islands. Different seeding layers like metals (Ag,^[^ ^72^ ^]^ Al,^[^ ^73^ ^]^ nickel (Ni),^[^ ^74^ ^]^ tin (Sn), Au,^[^ ^75^ ^]^ germanium (Ge),^[^ ^76^ ^]^ and Cu^[^ ^77^ ^]^), oxides (Tantalum pentoxide (Ta_2_O_5_),^[^ ^76^ ^]^ ZnO,^[^ ^78^ ^]^ nickel oxide (NiO),^[^ ^79^ ^]^ tellurium dioxide (TeO_2_),^[^ ^80^ ^]^ niobium pentoxide (Nb_2_O_5_),^[^ ^81^ ^]^ molybdenum trioxide (MoO_3_),^[^ ^82^ ^]^ and caesium carbonate (Cs_2_CO_3_)^[^ ^83^ ^]^), polymers (photoresist SU‐8,^[^ ^84^ ^]^ polyethyleneimine,^[^ ^85^ ^]^ and ormoclear^[^ ^86^ ^]^) and organic monolayers (11‐mercaptoundecanoic acid [MUA],^[^ ^87^ ^]^ [3‐aminopropyl]‐trimethoxysilane:[3‐ mercaptopropyl]‐trimethoxysilane [APTMS:MPTMS],^[^ ^88^ ^]^ methyl‐terminated alucone,^[^ ^89^ ^]^ and 1,4‐bis[2‐phenyl‐1,10‐phenanthrolin‐4‐yl]benzene [p‐bPPhenB]^[^ ^90^ ^]^) have been used to fabricate the ultrathin transparent metal films. In Ag‐based transparent films, using seed layers facilitates the development of high‐quality ultrathin film layers with roughness values of less than 0.5 nm (Cu as seed layer) and 1 nm (Au as seed layer). For example, in the study of Schubert et al.,^[^ ^73^ ^]^ the transparent film with 1 nm Au as the seed layer and 7 nm Ag layer resulted in a sheet resistance of 19 Ω sq^−1^ and transmittance of 83%, which are significantly better than Ag electrodes and even better than ITO electrodes.^[^ ^73^ ^]^ Ultrathin Ag‐based transparent films, produced using Cu and Au seed layers, demonstrate exceptional efficiency as counter electrodes in applications for organic solar cells (OSCs).^[^ ^91^ ^]^ They helped to enhance the efficiency of OSCs. Utilizing a sandwich structure comprising Ag metal film and Ge seed layers as electrodes in flexible organic light‐emitting diodes (OLEDs) yielded efficiency comparable to ITO‐based electrodes. Metal oxides and polymers‐based seed layers were also used to prepare ultrathin Ag films and to increase the mechanical and transmittance properties.^[^ ^92^ ^]^

Doping is a bulk strategy, where depositing a small amount of dopant and metal in the PVD process reduces the de‐wetting problem. The doping strategy is easier than the seeding strategy in terms of the fabrication process. Different dopants like Al,^[^ ^93^ ^]^ Cu,^[^ ^71^ ^]^ Ni,^[^ ^94^ ^]^ N,^[^ ^95^ ^]^ and O^[^ ^96^ ^]^ have been reported in the literature. A small amount of Al doping helped to decrease the Ag film percolation threshold from 20 to 6 nm.^[^ ^93^ ^]^ A 7 nm Al‐doped Ag film provided better conductivity (resistance of 28 ohm sq^−1^) and transparency (80%) as compared to pure Ag film.^[^ ^93^ ^]^ These films deposited on polyethylene terephthalate (PET) substrates showed excellent flexibility and durability compared to ITO.^[^ ^93^ ^]^ The amalgamation of seeding and doping techniques can potentially improve Ag film's properties by aiding in a more significant reduction of the percolation threshold.^[^ ^97^ ^]^ For example, Ta_2_O_5_/Al‐doped Ag electrodes in OSCs helped to achieve the same efficiency ITO‐based OSCs with better flexible properties.^[^ ^97^ ^]^

Cu is a better choice for making cost‐effective electrodes than Ag, which exhibits approximately similar properties.^[^ ^98^ ^]^ The nitrogen (<1%) helps to form a continuous ultrathin Cu film, and an N‐doped Cu film can exhibit a resistance of 20 ohms sq^−1^ and a transparency of 84%.^[^ ^98^ ^]^ However, oxidation is one major issue with Cu‐based metal films. So, to address the oxidation issues, researchers developed a sandwich‐structured Cu film with two sides coated with oxides like ZnO.^[^ ^98^ ^]^ Utilizing a sandwich‐based structure for Cu‐based films has effectively mitigated the oxidation issues associated with Cu‐based electrodes, rendering them more reliable and stable for various applications.

Metal nanowires (MNWs) are a promising form of FTE materials, mainly due to their high chemical stability, solution processability, diversity of bulk synthesis methods, higher transmittance, and flexibility compared to film layers.^[^ ^99^ ^]^ Various MNWs, such as Ag, Au, and Cu, have been widely used to fabricate transparent electrodes.^[^ ^100^, ^101^, ^102^ ^]^ An optically transparent and electrically conductive percolation network can be created by controlling the porosity and thickness of transparent electrodes. Furthermore, the metal nanowires' network structure and intrinsic flexibility contribute to the electrode's softness.^[^ ^103^ ^]^ In addition to the benefits of transparent electrodes based on MNWs, challenges such as contact resistance between wires and the presence of organic content in MNW solutions impact the conductivity of these transparent electrodes. Even though the individual nanowires possess excellent conductivity of 6 × 10^7^ S m^−1^, the sheet resistance of MNWs‐based FTEs is usually in the range of 10^4^–10^6^ Ω sq^−1^ due to the presence of organics like polyvinyl pyrrolidone (PVP) and the loose contact wire to wire junctions.^[^ ^104^ ^]^ This high resistance is far less effective compared to ITO‐based electrodes and hence cannot meet the requirements of applications. To overcome the resistance issues in MNW‐based transparent electrodes, researchers have investigated composites that incorporate MNWs with materials such as graphene, reduced graphene oxides,^[^ ^105^, ^106^, ^107^ ^]^ or conductive polymers like PEDOT.^[^ ^108^ ^]^ These composite materials have been effective in reducing the resistance of MNW‐based transparent electrodes by improving the welding of nanowire junctions or providing additional pathways for carrier transport.^[^ ^1^, ^109^ ^]^ For example, the resistance of Ag nanowire‐based transparent electrodes was reduced from 1.5 × 10^4^ Ω sq^−1^ to 100 Ω sq^−1^ by adding graphene oxide, which improved junction welding and carrier pathways.^[^ ^1^ ^]^ The high roughness is another big challenge in fabricating MNW‐based transparent electrodes. Typically, nanowires with a 90 nm diameter are used to fabricate transparent electrodes. Consequently, stacking three nanowires at a specific junction results in a maximum peak‐to‐valley value of 270 nm, posing challenges for device applications.^[^ ^110^, ^111^ ^]^ Various approaches, including the utilization of conducting and nonconducting polymers and the implementation of hot‐pressing strategies, have been explored to reduce the roughness of transparent electrodes based on metal nanowires to below 20 nm.^[^ ^112^, ^113^ ^]^ The intricate synthesis procedures and the spray fabrication process involved in MNWs‐based flexible transparent electrodes pose an additional challenge, contributing to the high cost of manufacturing these electrodes. So, further reducing manufacturing costs and increasing productivity have been achieved by incorporating the high‐throughput roll‐to‐roll technique into the coating process.

Metal mesh is another structure of metal‐based FTEs. Periodically or randomly transparent and conductive metal meshes are patterned on flexible substrates for metal mesh‐based thin film structures.^[^ ^104^, ^114^ ^]^ In contrast to metal nanowire networks, metal meshes are not subject to wire‐to‐wire contact resistance or high haze levels. In addition, the linewidth, thickness, pitch, and pattern of metal meshes can be easily adjusted to optimize the balance between sheet resistance and optical transmittance.^[^ ^1^ ^]^ However, it is usually challenging to fabricate metal mesh‐based FTEs because they typically involve expensive technologies such as, nanoimprint lithography and metal etching processes.^[^ ^115^, ^116^ ^]^ Furthermore, they are also subject to the high surface roughness (8–20 nm) that occurs similar to metal nanowire networks.^[^ ^117^ ^]^ For instance, in a particular study, nickel electrodeposition was employed on Ag‐based mesh electrodes to reduce electrode roughness to 0.17 nm, yielding superior results compared to ITO electrodes.^[^ ^118^ ^]^ Consequently, researchers have been developing simple, low‐cost, and scalable methods like solution‐based electrodeposition, site‐selective electrodeposition, and the combination of lithography, electrodeposition, and imprint transfer for fabricating metal mesh‐based FTEs with low surface roughness in this field.^[^ ^119^, ^120^, ^121^ ^]^

Metal nanowires, including Ag and Au, are used for their superior conductivity and flexibility. AgNWs are often integrated with polymers like PEDOT to enhance performance.^[^ ^122^ ^]^ While AgNWs exhibit excellent electrical properties, their biocompatibility is a concern due to potential cytotoxicity from silver ions.^[^ ^123^ ^]^ Encapsulation with biocompatible polymers or the use of gold nanowires, which are generally considered more biocompatible, are strategies to mitigate these risks.

### 2.3 Conductive Polymer‐Based Transparent Electrode Materials

Conductive polymers, have emerged as alternatives to traditional transparent electrodes, combining optical transparency, electrical conductivity, and mechanical flexibility. These materials offer a range of advantages, making them popular in various applications, from optoelectronic devices to biomedical sensors.^[^ ^98^, ^101^, ^102^ ^]^ Table S3 (Supporting Information) shows detailed information about the conductive polymer‐based transparent electrode materials and their different applications and advantages from the literature.

The unique combination of properties found in conductive polymers like PEDOT: PSS is particularly beneficial. Their adaptability is evident in applications such as transparent neural electrode arrays,^[^ ^115^ ^]^ inkjet‐printed electrode arrays,^[^ ^116^ ^]^ and organic light‐emitting electrochemical cells,^[^ ^118^ ^]^ with each application highlighting a different aspect of these materials' versatility (Figure 2d,e). Integrating PEDOT: PSS with materials like silver nanowires significantly broadens its application spectrum, making it suitable for diverse uses, including in optoelectronic devices, biomedical sensors, flexible electronics, energy storage solutions, and as transparent electrodes.^[^ ^33^, ^66^, ^124^, ^125^ ^]^

PEDOT:PSS in different composite forms, has also been used for OSC applications. These flexible solar cells are popular due to their low cost, portability, lightweight, and ease of fabrication.^[^ ^126^, ^127^, ^128^, ^129^, ^130^ ^]^ For instance, in hybrid solar cell based on solution processes graphene oxide welded silver nanowires have been utilized to prepare transparent conductive electrodes. These sandwich structures containing AgNW network embedded in PEDOT:PSS and graphene oxide show high power conversion efficiency, with a reported efficiency of 13.3%. The developed electrode shows a sheet resistance of 13.3 Ω sq^−1^ and a transmittance of 82.2%, which is comparable to the commercial ITO glass.^[^ ^131^ ^]^ Furthermore, a flexible perovskite solar cell has been developed using solution‐processed transparent polymeric film.^[^ ^132^ ^]^ In the study of Zhu et al.,^[^ ^132^ ^]^ PEDOT:PSS treated with thin‐film iodide, a nonacidic agent, improved its electrical conductivity from 0.3 to 1000 S cm^−1^ compared to the pristine PEDOT:PSS thin film. These films have excellent optical transparency with a transmittance of 85% from 350 to 550 nm and 75% transmittance from 550 to 900 nm, reporting a power conversion efficiency of 13.36%. This study provides an easy route to develop flexible perovskite solar cells.^[^ ^132^ ^]^ In addition, a nonfullerene OSC using solution‐processed PEDOT:PSS doped with trifluoromethanesulfonic acid has been reported.^[^ ^133^ ^]^ This polymeric thin film possesses a low sheet resistance of 35 Ω sq^−1^, high electrical stability, and superior wettability. This flexible organic solar cell has a high‐efficiency yield of 16.41% and can perform 1000 cycle bending tests at a 1.5 mm radius, demonstrating high flexibility and good thermal stability at 85 °C.

Despite their significant advantages, conductive polymers pose challenges when used as transparent electrodes. Intrinsically conducting polymers like polyaniline (PANI),^[^ ^134^ ^]^ polypyrrole (PPy),^[^ ^135^ ^]^ and polythiophene (PTh),^[^ ^136^ ^]^ while electrically conductive, often lack sufficient optical transmittance. This drawback prevents their applications in areas requiring high transparency and conductivity, such as display technologies, light‐emitting devices, and photovoltaic panels, where clear visibility and efficient light management are paramount.^[^ ^137^, ^138^, ^139^ ^]^ This limitation necessitates the exploration of alternative materials and approaches.

In response to these challenges, extensive research and development have been conducted to enhance the efficiency of conductive polymers as TEs.^[^ ^140^ ^]^ Toward this aim, integrating conductive fillers, such as metal nanowires, CNTs, and graphene, into the polymer matrix has been a significant advancement.^[^ ^141^ ^]^ For instance, incorporating a low volume of CNTs (0.0068–0.068 vol.%) into polymethyl methacrylate (PMMA) yielded transparent and electrically conductive CNT‐PMMA composite pellets.^[^ ^142^ ^]^ These composites showed an optical transmittance of up to 65% with just 0.0068 vol.% CNT, compared to the 90% transmittance of pure PMMA pellets. Concurrently, the electrical conductivity of these composites was enhanced, ranging from 10^−5^ to 10^−1^ S m^−1^ with CNT volumes of 0.0068–0.045 vol.%.^[^ ^142^ ^]^

Another study reported the preparation of electrically conductive, highly transparent, and flexible thin films based on copolyamide filled with up to 5 wt.% titanium carbide Ti_3_C_2_T_x_ (MXene) nanosheets.^[^ ^143^ ^]^ These films retained a high transparency level, exceeding 75%, even with 5 wt.% MXene content. The electrical conductivity of these composites reached 1.4 × 10^−2^ S cm^−1^ when filled with 5 wt.% (1.8 vol.%) of MXene.^[^ ^143^ ^]^ This relatively high electrical conductivity and suitable transparency position these materials as promising candidates for flexible electronics applications. These approaches improve the electrical conductivity of the polymers and maintain their inherent transparency.

Recent developments in conductive polymers have demonstrated their potential in various applications, from enhancing optoelectronic devices with light/color adjustability and electromagnetic protection to creating advanced electrodes for flexible transparent electronics.^[^ ^144^ ^]^ Creating hybrid systems, such as the AgNW–graphene oxide hybrid electrode, exemplifies the fusion of conductive polymers with other materials.^[^ ^101^ ^]^ Graphene oxide offers high mechanical strength, excellent transparency, and chemical stability, while silver nanowires provide high electrical conductivity and flexibility.^[^ ^145^ ^]^ This combination ensures that the hybrid electrode maintains its performance under mechanical deformation and provides efficient electrical and optical properties needed for smart window applications. This hybrid electrode combines the conductivity of Ag NWs with the flexibility of graphene oxide and finds applications in smart windows (Figure 2f). Such combinations have enhanced electrical and optical properties, paving the way for new applications.^[^ ^146^, ^147^ ^]^ Furthermore, developing solution‐processed methods for PEDOT:PSS transparent conductive thin films, represents a significant advancement,^[^ ^148^ ^]^ this method eliminates the need for post‐treatment, reducing waste and fabrication time. In organic solar cells, the use of conductive polymers has shown significant potential, as presented in Figure 2g.^[^ ^149^ ^]^ Additionally, conductive hydrogels have gained attraction in flexible electronics. Conductive hydrogels are particularly useful in triboelectric nanogenerators due to their flexibility, high ionic conductivity, and ability to form conformal contact with various surfaces, enhancing the efficiency of energy harvesting.^[^ ^150^ ^]^ The inherent stretchability and biocompatibility of hydrogels make them ideal for wearable devices that require flexible energy sources. A notable development in this area is the creation of a transparent, freezing‐tolerant organ hydrogel that can be used as a strain sensor and in triboelectric nanogenerators, showcasing the versatility of these materials (Figure 4e).^[^ ^151^ ^]^

In the context of smart windows, hybrid electrodes composed of silver nanowires and graphene oxide are highly effective.^[^ ^152^ ^]^ Silver nanowires offer exceptional conductivity, while graphene oxides contribute corrosion resistance against the electrolyte used in smart windows.^[^ ^153^ ^]^ The percolation network of Ag nanowires combined with the 2D structure of graphene oxides ensures outstanding mechanical properties, including flexibility. These developments indicate the potential of conductive polymers in various advanced technological applications. However, challenges related to their stability, biocompatibility, and fabrication remain.

The advancements in conductive polymer‐based transparent electrodes highlight the rapid progress in this domain. While these materials offer numerous advantages, ongoing research and development are critical in addressing the remaining challenges, the presence of residual monomers or additives can affect biocompatibility, necessitating thorough purification and characterization.^[^ ^154^ ^]^ As the field continues to evolve, conductive polymers are anticipated to play an increasingly crucial role in the future of transparent electrodes, contributing significantly to various technological advancements.

### 2.4 Graphene and Carbon‐Based Transparent Electrode Materials

Carbon nanomaterials (CNs) have many advantages over alternatives like ITO. They're cost‐effective, environmentally friendly due to abundant carbon resources, and more durable. CNs also offer excellent electrical properties, flexibility, and biocompatibility, making them ideal for innovative applications like wearable electronics,^[^ ^155^ ^]^ rollable displays,^[^ ^156^ ^]^ and electronic textiles.^[^ ^157^ ^]^ Studies have demonstrated that graphene and CNTs can support cell growth and differentiation, making them suitable for biosensors and tissue engineering.^[^ ^158^, ^159^ ^]^ However, the potential for oxidative stress and inflammatory responses requires careful assessment of their surface chemistry and long‐term interactions with biological tissues. Various CNs, such as CNTs and graphene derivatives, are commonly used in transparent carbon electrodes (TCEs).^[^ ^1^, ^160^ ^]^ Table S4 (Supporting Information) lists graphene and carbon‐based transparent materials, offering insights into their properties and applications.

1D semiconducting materials, such as CNTs, are widely used in conductive transparent electrodes due to their unique structures. CNTs come in single‐walled (SW‐SNTs), double‐walled (DW), or multi‐walled (MWCNTs) hollow carbon cylinders, with variations in length, diameter, and chirality.^[^ ^161^ ^]^ Their exceptional characteristics are excellent electrical conductivity, high optical transparency, substantial surface area, and chemical and thermal stability. They make CNTs promising for transparent conductive films.^[^ ^162^, ^163^, ^164^ ^]^ CNTs' extended π‐system allows for direct charge transport with exceptional mobility, and extensive research efforts have focused on their applications.^[^ ^164^ ^]^

CNT‐based transparent conductive films (TCFs) have a significant advantage over alternatives like ITO, PEDOT: PSS, graphene, and Ag grids due to low optical absorption.^[^ ^165^ ^]^ In the visible and near‐infrared (NIR) range, CNT and graphene films offer similar transmittance, while ITO and PEDOT decline in transmittance beyond 1000 nm (Figure 3c).^[^ ^166^ ^]^ Ag grids become more reflective with increasing wavelength, reducing NIR transmittance.^[^ ^167^ ^]^ CNTs, with low charge carrier density, are suitable for IR solar cells, providing transparency beyond 1000 nm.^[^ ^168^ ^]^

The electrical conductivity of CNT‐based transparent conductive films demonstrates superior performance in comparison to other types of TCFs, such as those composed of metal oxides, as illustrated in Figure 3d.^[^ ^163^ ^]^ Researchers often use the percolation system to study the transport behavior of thin CNT networks.^[^ ^169^ ^]^ Beyond the percolation threshold, CNT films' resistance (Rs) decreases significantly as CNT density or surface coverage increases. CNT alignment and bundle length also play a crucial role in deciding the transport properties of CNT‐based TCFs. Randomly distributed CNT films are preferred due to their lower threshold, offering a clearer conductive path compared to perfectly aligned CNTs.^[^ ^170^, ^171^ ^]^ In CNT films, resistance arises from CNT junction contacts. Longer CNTs are preferred for TCF fabrication as they reduce junction resistance (Figure 3e).^[^ ^172^, ^173^, ^174^ ^]^ The diameter of CNT is also an important parameter for the superior conductivity of TCFs; for example, single‐walled carbon nanotubes (SWCNTs) with larger diameters (1.4 nm) lead to higher sheet conductance in films, particularly in semiconducting SWCNTs with smaller band gaps due to their high charge carrier density (Figure 3f).^[^ ^169^, ^175^, ^176^, ^177^ ^]^ Another parameter that affects the conductivity of TCFs is the purity of CNTs. The presence of amorphous carbon in the form of oxygen functional groups affects the conductivity.^[^ ^178^ ^]^ CNT synthesis methods are critical for TCF resistance. Arc discharge is an efficient method,^[^ ^179^ ^]^ while alternatives like chemical vapor deposition (CVD), laser ablation, and template synthesis also exhibit good conductivity (Figure 4f).^[^ ^179^, ^180^, ^181^, ^182^ ^]^ In summary, the conductivity of CNT‐based TCF is influenced by several factors such as CNT length, CNT diameter, M/S (Metal‐to‐Semiconductor) ratio, alignment, and purity. A trade‐off exists between optimizing TCF transport properties and keeping the synthesis process simple and cost‐effective when moving toward commercial use.

Graphene, with its remarkable properties like high electrical conductivity (200 000 cm^2^ V^−1^s^−1^), excellent transparency (97.4%), and mechanical strength (Young's modulus = 1 TPa), has garnered significant attention as a transparent electrode material.^[^ ^183^, ^184^, ^185^, ^186^ ^]^ Compared to other carbon‐based materials for TCFs, graphene‐based TCFs are highly competitive and extensively studied. In 2010 and 2013, large scale roll‐to‐roll fabrication of graphene‐based flexible TCEs has been demonstrated by Samsung and Sony for their use in electronic displays,^[^ ^187^, ^188^ ^]^ leading to a surge in research on graphene‐based transparent conductors (Figures 3g and 4f,g). Flexible TCEs will be the first industrial application field for graphene.^[^ ^187^ ^]^

Graphene‐based transparent electrodes are a top choice for flexible and wearable electronics, replacing ITO‐based TCFs. Graphene offers high optical transmittance, excellent carrier mobility, mechanical flexibility in single‐layer graphene, and minimal light absorption (only 2.3%).^[^ ^189^ ^]^ While graphene holds promise for innovative transparent conductors, there's room for improving the electrical performance of graphene‐based networks to meet the demands of wearable applications and replace ITO.

Various surface modification strategies enhance the optoelectronic properties of graphene‐based TCFs. One effective strategy is chemical doping, which increases carrier concentration and reduces sheet resistance in transparent electrodes while maintaining or improving light transmittance (Figure 3h).^[^ ^166^, ^180^ ^]^ Common p‐type dopants like nitric acid (HNO_3_), thionyl chloride (SOCl_2_), thionyl bromide (SOBr_2_), iron(III) chloride (FeCl_3_), and gold(III) chloride (AuCl_3_) significantly enhance the electrical conductivity of graphene‐based TCEs.^[^ ^190^ ^]^ However, the environmental stability of doped graphene‐based TCEs can be a concern as doping molecules may desorb from graphene sheets, increasing sheet resistance. Graphene sheet size also plays a crucial role in improving the conductivity of graphene‐based TCEs. Larger graphene sheets improve conductivity in transparent electrodes by reducing contact resistance, as shown in Figure 3i.^[^ ^191^, ^192^ ^]^ Efforts to obtain large graphene sheets have focused on derived graphene oxide (GO).^[^ ^193^, ^194^, ^195^ ^]^ Liquid‐phase exfoliated graphene sheets are challenging to enlarge due to long‐term sonication, typically having a lateral size of less than 2 µm. Enhancing electrical conductivity is practical by removing surfactants and organic solvents from the graphene surface from liquid phase exfoliated graphene.^[^ ^196^ ^]^ The conductivity of graphene‐based TCEs can be improved by combining 1D conductive materials with 2D graphene. Combining these materials resolves inter‐sheet junction resistance as the conductivity of graphene‐based TCEs improves.^[^ ^197^ ^]^ The hybrid method involving 1D CNTs, metal nanowires, and graphene effectively bridges sheets, reduces surface roughness, and enhances optoelectronic properties, outperforming ITO (Figures 2h and 4h).^[^ ^198^, ^199^, ^200^ ^]^ Another approach involves adding graphene to the conductive polymer PEDOT: PSS (Figure 4i).^[^ ^201^, ^202^ ^]^ The resulting graphene/PEDOT hybrid TCEs offer low sheet resistance, high light transparency, and excellent mechanical and electrical stability,^[^ ^203^ ^]^ surpassing ITO in OLED performance and making them ideal for wearables. Conductive polymers also reduce surface roughness and prevent short circuits.^[^ ^204^ ^]^

In addition to enhancing the fundamental characteristics of graphene‐based transparent electrodes, the processing methods for these transparent electrodes are contingent upon their intended applications. Consequently, distinct processing techniques for various transparent electrodes are formulated based on the specific application requirements. Graphene‐based TCFs are crucial in applications like stretchable displays, epidermal sensors, OLEDs, and solar cells, where electrical conductivity must be maintained during mechanical deformations.^[^ ^205^ ^]^ Simply producing high‐quality graphene is not enough; efficient fabrication methods are needed to create an elastic conductive graphene network. This structure enables flexible and stretchable conductive paths on polymer substrates, ensuring exceptional electrical stability during mechanical stretching.^[^ ^206^ ^]^ Different mechanisms have been used for graphene‐based TCFs to work efficiently as stretchable transparent electrodes. The mechanisms for graphene network design include: i) Graphene‐based TCEs with stretchable patterning,^[^ ^207^ ^]^ ii) 1D graphene nano scrolls bridging 2D sheets,^[^ ^208^ ^]^ iii) tailored graphene‐based TCEs in a stretchable mesh,^[^ ^209^ ^]^ and iv) graphene‐based TCEs with wrinkled/wavy structures for increased stretchability.^[^ ^210^ ^]^

## 3 Multimodal Sensing Applications

Multimodal sensing is the capability of simultaneous detection of different types of stimuli applied to the sensors. It utilizes multiple sensing mechanisms integrated into a single device. Multimodal sensing applications have been studied significantly in recent years since multimodal sensing devices provide more comprehensive and accurate information by integrating multiple sensing modalities into a single device.^[^ ^211^, ^212^, ^213^, ^214^, ^215^, ^216^ ^]^ These sensing modalities can include: 1) optical and electrical sensing: used for neural imaging,^[^ ^217^ ^]^ skin‐like soft sensor applications,^[^ ^13^ ^]^ and solar cells application^[^ ^132^ ^]^; 2) optical and acoustic sensing: applied in wireless communications for wearable systems,^[^ ^218^ ^]^ tumor imaging,^[^ ^219^ ^]^ cardiovascular monitoring^[^ ^220^ ^]^; 3) optical and mechanical sensing: utilized in photoacoustic imaging,^[^ ^221^, ^222^ ^]^ electromechanical sensor^[^ ^223^ ^]^; and 4) quantum sensing: employed for magnetic field imaging,^[^ ^224^ ^]^ cellular activity monitoring,^[^ ^216^, ^225^, ^226^ ^]^ neuroimaging and diagnosis of heart disease,^[^ ^227^, ^228^ ^]^ disease like malaria.^[^ ^229^, ^230^ ^]^ These devices find applications in various fields, including healthcare for patient monitoring,^[^ ^231^, ^232^ ^]^ environmental sciences for detecting various environmental parameters,^[^ ^233^, ^234^, ^235^ ^]^ and wearable electronics for user interaction and health monitoring.^[^ ^236^, ^237^, ^238^, ^239^ ^]^

Transparent electrode materials play a critical role in the functionality of multimodal sensors. Their transparency allows for optical observation or measurements, while their electrical conductivity enables electric or electrochemical sensing. For instance, in healthcare applications, transparent electrodes enable simultaneous optical imaging and electrical signal recording, providing a more comprehensive understanding of physiological processes. In environmental monitoring, these materials can facilitate the detection of both optical and electrochemical signals, enhancing the sensor's sensitivity and selectivity. However, the choice of transparent electrode material can significantly impact the sensor's performance, necessitating careful consideration of the material's optical, electrical, and mechanical properties. Based on the material properties, the application of multimodal sensing is categorized as optoelectrical, optoacoustic, optomechanical, and quantum sensing; these are discussed in detail in the below section:

### 3.1 Optoelectrical Sensing

Optoelectronic devices serve as converters between electrical and optical signals, utilizing these conversions in their functions.^[^ ^240^ ^]^ Transparent electrodes with their outperforming electrical conductivity and optical transparency, are indispensable in many optoelectronic devices. For the past few decades, ITO and FTO have mainly dominated these roles. With the advancement in the organic transparent electrodes, PEDOT:PSS, carbon nanotubes, metal nanowire networks, and graphene have also been employed in optoelectronic devices.^[^ ^51^, ^241^ ^]^ These materials offer enhanced photoelectric properties, such as high optical transparency and electrical conductivity, making them suitable for applications requiring simultaneous optoelectrical measurements.^[^ ^242^ ^]^ PEDOT:PSS‐based transparent electrodes demonstrate enhanced electrical conductivity and stretchability, making them ideal for wearable sensors that monitor physiological signals like electrooculogram, electrocardiogram, and electromyogram with high signal‐to‐noise ratios.^[^ ^243^ ^]^ Additionally, poly(lactic‐co‐glycolic acid) (PLGA) and molybdenum‐based transparent electrodes enable bidirectional cardiac interfacing by providing multiparametric electrical/optical mapping of cardiac dynamics.^[^ ^244^ ^]^

Transparent photoelectric devices using metal oxides, such as NiO/ZnO junctions, exhibit remarkable properties for UV photodetection with high transparency and excellent responsivity. These devices efficiently absorb UV light while allowing the transmission of visible light, making them strong candidates for applications in flame detection and missile defense. For example, a NiO/ZnO transparent photodetector has been shown to provide high transparency (≈90%) and strong protection against UV exposure, with a responsivity of 3.85 A W^−1^ and detectivity of 9.6 × 10^13^ Jones, demonstrating fast UV photodetection performance with a rise time of 24.2 ms.^[^ ^245^ ^]^ Another highly stretchable transparent electrode based on a protic ionic liquid with PEDOT: PSS and, 3‐methylimidazolium:bis(trifluoromethylsulfonyl)amide (p‐MIM:TFSI) has been reported.^[^ ^13^ ^]^ High water miscibility of p‐MIM and its favorable ion exchange capability with PEDOT leads to a better electrical conductivity (σ = 450 S cm^−1^) and thin‐film stretchability of these electrodes, with a crack onset strain (εc) exceeding 50%.^[^ ^13^ ^]^ These PEDOT/p‐MIM layers are biocompatible and can adhere to human skin, enabling the collection of multimodal signals, including electrooculogram, electrocardiogram, and electromyogram. The penetration of light through human skin during biological activity to record stable signals presents an opportunity for the everyday use of wearable signal sensors applied to exposed human skin.^[^ ^13^ ^]^

**Figure 5:**
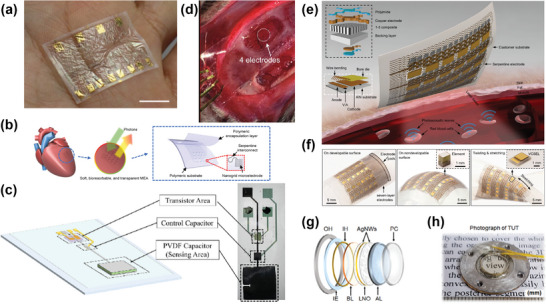
a) A fully transparent ultrathin OECTs conformable to the skin (scale bar 1 cm),^[^ ^192^ ^]^ b) Schematic illustration of microelectrode arrays (MEA) on a heart. The device consists of Mo nano grid microelectrodes and interconnects, PLGA encapsulation, and substrate layers.^[^ ^244^ ^]^ c) Photo and sketch of the integrated vertical channel OCMFET for multimodal tactile sensing. The transistor area, the control capacitor, and the PVDF capacitor glued on the floating gate are highlighted. (Scale bar 1 mm).^[^ ^246^ ^]^ d) Photograph of transparent and flexible ECoG EA fabricated with AgNWs20 + IZO films placed over the cortex of an anesthetized rat. Reprinted with permission from ref. [59]. Copyright 2021 American Chemical Society. e) Schematics of the device structure and the working principle. The patch comprises an array of VCSELs and an array of piezoelectric transducers, interconnected by serpentine copper electrodes, and f) photographs of the soft photoacoustic patch under different modes of deformation, including bending on a developable surface, wrapping on a nondevelopable surface, and stretching and twisting. Insets in the middle and right panels are micrographs of a single transducer element and a VCSEL diode, respectively.^[^ ^249^ ^]^ g,h) Photograph demonstrating the transparency of a TUT with an element size of 9 mm.^[^ ^219^ ^]^

Integrating Ag nanowires with PEDOT:PSS, a conducting polymer, has been used to develop flexible, fully transparent, ultrathin, organic electrochemical transistors (OECTs) for human stress monitoring (**Figure** **5**a).^[^ ^192^ ^]^ The flexible OECTs have visible transmittance of >90% and transconductance of ≈1 mS at operations of <0.6 V.^[^ ^192^ ^]^ Transparent OECTs allow the simultaneous measurement of optical blood flowmetry along with electroencephalogram acquisition, and nitrate ion sensing, due to their transparency.^[^ ^192^ ^]^ For optical assessment, electroencephalogram signals were recorded with open and closed eyes, and these signals were introduced to the OECTs gate, including drain–current modulation. A wireless module was used to detect the voltage signals of a serial resistor. During closed eyes, alpha rhythms were acquired, indicating a relaxed state of mind, while these signals weakened when the eyes were open. These feasibility demonstrations suggest a potential for human stress monitoring in bioelectronics.^[^ ^192^ ^]^

PLGA and molybdenum‐based fully bioresorbable, soft, and transparent microelectrode array platform was used for bidirectional cardiac interfacing. Figure 5b shows a schematic representation of a 4 × 4 microelectrode array (MEA) platform attached to the heart. The developed MEAs showcased their effectiveness using both ex vivo and in vivo cardiac models by measuring three key cardiac parameters (Electrogram (EG), Transmembrane Potential (Vm), and Cytosolic Calcium (Ca^2+^)) through various imaging modalities. This allowed the precise detection of cardiac wave propagation patterns and the assessment of electrical and mechanical dynamics. These electrodes enable electrical and optical mapping and provide targeted site‐specific pacing to study and address cardiac dynamics, cardiac dysfunctions in different heart models such as rat and human. The bioresorbable device leverages its potential use in postsurgical monitoring after ischemia, myocardial infarction and treatment of temporary pathological conditions in patients after transcatheter aortic valve replacement.^[^ ^244^ ^]^

A flexible Organic Charge Modulated Field Effect Transistor (OCMFET)‐based highly sensitive multimodal tactile transducer has been reported for simultaneous detection of temperature and force.^[^ ^246^ ^]^ The device's unique structure allows tuning sensitivity by changing its geometrical design parameters, while the vertical features enable short‐channel, miniaturized organic transistors. The device was fabricated as an integrated sensor with a polyvinylidene fluoride (PVDF) capacitor with an area of 0.25 cm^2^ (Figure 5c). The integration of the short‐channel charge sensor and pyro/piezoelectric sensing element allows multimodal tactile sensor to transduce temperature and force variations with improved sensitivity and compact size.^[^ ^246^ ^]^

A recent study reported the development of cost‐effective and high‐performance ECoG electrodes using hybrid films composed of AgNWs and IZO.^[^ ^59^ ^]^ The resulting transparent films exhibited a sheet resistance of 6 Ω sq^−1^. An impedance of 20 kΩ at 1 kHz and a charge storage capacity of 3.2 mC cm^−2^ was achieved using electrodes with a 500 µm diameter, showcasing improved properties compared to IZO electrodes, which perform similarly to classical ITO. Light‐induced artifact characterization demonstrated that light intensities below 14 mW mm^−2^ induced minimal electrical potential variation, falling within the range of baseline noise. The effect of high network density on the distortion of light during fluorescence microscopy was studied using a film of AgNWs + IZO on a coverslip, covered with brain slices expressing mCherry in the neurons and the fluorescent dye in all neurons. Confocal images concluded that the AgNWs + IZO film does not affect optical imaging. Recording the neural activity from the rat brain was used for in vivo validation.^[^ ^59^ ^]^ Figure 5d illustrates the placement of electrodes and the craniotomy, highlighting the capabilities of the system. Notably, the hybrid films did not distort light during imaging. The study emphasized the effectiveness of developed transparent ECoG electrodes.^[^ ^59^ ^]^ By utilizing accessible and cost‐effective techniques and materials, this research aimed to simplify production, providing a scalable and economical tool for researchers to record neural electrical activity on a large scale.^[^ ^59^ ^]^

### 3.2 Optoacoustic Sensing

Optoacoustic sensors or photoacoustic sensors can generate ultrasound waves by absorbing light due to the thermoelastic expansion.^[^ ^247^ ^]^ Chen et al.^[^ ^248^ ^]^ developed a transparent ultrasound transducer (TUT) array capable of real‐time quad‐mode ultrasound, photoacoustic, Doppler ultrasound, and fluorescence imaging, directly connected to tissue‐mimicking phantoms.^[^ ^248^ ^]^ The array, consisting of 64 elements and centered at ≈6 MHz, was used for real‐time ultrasound (US) and photoacoustic (PA) imaging with the TUT‐array connected to the data acquisition system on a tissue phantom. The phantom contained four metal wire targets, each with a 50 µm diameter, dyed with India ink for strong photoacoustic contrast, and two hypoechoic regions filled with agar solution to mimic tissue. The TUT‐array was tested on a phantom and laser light was used to demonstrate dual‐modality ultrasound and photoacoustic (USPA) imaging with minimal coupling of the array. The same imaging was performed using a commercial linear probe (ATL L7‐4, Philips) for comparison. The USPA imaging revealed the four micrometal wire targets, which had different acoustic impedances. The measured sizes of the imaged regions matched the tube diameter of ≈2 mm. This array shows potential for multimodal imaging applications in diagnosing cancer, vascular diseases, neurological conditions, and for image‐guided endoscopy and wearable imaging.^[^ ^248^ ^]^ A soft photoacoustic patch with an array of ultrasonic transducers made of a piezoelectric layer of 2 MHz lead zirconate titanate (PZT) micropillars and vertical‐cavity surface‐emitting laser (VCSEL) diodes have been developed for 3D hemoglobin mapping in deep tissues.^[^ ^249^ ^]^ The patch involves rigid components but having an overall soft character. (Figure 5e). The high‐power VCSEL diodes emits laser pulses that penetrate over 2 cm into biological tissues. This activates the hemoglobin molecules, producing acoustic waves, captured by the transducers creating high‐resolution 3D image of hemoglobin (Figure 5f). The linear relationship between photoacoustic signal amplitude and temperature allows accurate and fast 3D core temperature mapping. In vivo feasibility was demonstrated by imaging veins in the forearm, hand, and foot to monitor venous response during occlusion tests. The images were converted to 3D images displaying the vein structure of different body locations. The image of the internal jugular vein (IJV) in the neck was also captured ≈2 MHz, resulting in the low contrast of the vein in the ultrasound B‐mode image. This technology, accessing biomolecules in deep tissues, enhances wearable electronics capabilities and has significant implications for research and clinical applications.^[^ ^249^ ^]^ Noninvasive, multimodal photoacoustic tomography and optical coherence tomography (PAT/OCT) scanner has been used to 3D in vivo imaging of skin.^[^ ^250^ ^]^ This system employs an integrated optical detection scheme for both modalities using a shared 2D optical scanner. A Fabry–Perot polymer film ultrasound sensor, transparent with a spectral range of 590–1200 nm, detected photoacoustic waves on the skin surface, allowing both photoacoustic excitation and OCT probe beams to transmit through the sensor and into the tissue. The OCT has an axial resolution of 8 µm with lateral resolutions of 18 µm, while PAT achieved axial resolutions 20 µm, and lateral resolution 50–100 µm. The improved imaging depth, thanks to the longer wavelength OCT beam (1050 nm), provided high‐resolution 3D images of the vasculature and surrounding tissue up to 5 mm depth for different skin characterization applications such as burns, wounds, dermatitis, and other superficial tissue abnormalities.^[^ ^250^ ^]^

A single‐crystal transparent ultrasound transducer and a quadruple fusion imaging system based on lithium niobate have been developed.^[^ ^219^ ^]^ This system uses a spherically focused transparent ultrasound transducer to integrate ultrasound imaging with OCT, photoacoustic imaging, and fluorescence imaging. The transducer is fabricated with different conductive and supportive layers (Figure 5g,h). This imaging system was utilized for two applications: monitoring multiparametric responses to chemical and suture injuries in rat eyes, including corneal neovascularization and structural changes, and in vivo imaging of tumors, using Polyethylene Glycol (PEG)ylated gold nanorods. This integrated multimodal system is suitable for ophthalmology, oncology, and other healthcare applications with significant impact.^[^ ^219^ ^]^

**Figure 6:**
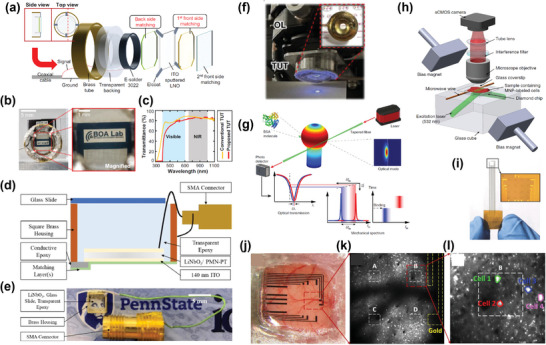
a) Structural schematic of the TUT, b) demonstration of the optical clarity of the TUT, and c) Measured optical transmittance of the proposed TUT.^[^ ^251^ ^]^ d) A schematic showing the TUT. The matching layer can be composed of parylene, or parylene + glass slide, and e) a photograph of a fabricated 3 mm × 3 mm TUT. Reprinted with permission from ref. [252], f) Photograph of the UV‐TUT, demonstrating its UV transparency. The red dashed box outlines a photograph of the UV‐TUT, showing an image passing through the UV‐TUT^[^ ^253^ ^]^ © 2023 Wiley‐VCH GmbH. g) Schematic illustrating the sensing mechanism. A protein molecule bound to an optomechanically oscillating microsphere yields an optical resonance shift δλ, which is transduced to a mechanical frequency shift δfm. The color map on the microsphere shows the radial breathing mechanical mode simulated by the finite element method.^[^ ^254^ ^]^ h) Wide‐field NV diamond magnetic imaging microscope. Samples containing immunomagnetically labeled cells are placed directly on the surface of a diamond chip with a highly enriched surface layer of NV centers. NV electronic spins are probed by optically detecting magnetic resonance using 532‐nm laser light and microwaves, with NV fluorescence imaged onto a scientific complementary metal‐oxide semiconductor (sCMOS) camera. For each imaging pixel, this procedure determines the magnetic field projection along one of the diamond axes over a 1 mm × 0.6 mm field of view. Reprinted with permission from ref. [225], i) A photo of the array. The inset is a microscopic image of the 4 × 4 array of the SU‐8 openings^[^ ^270^ ^]^ © 2018 WILEY‐VCH Verlag GmbH & Co. KGaA, Weinheim. j) Simultaneous in vivo calcium imaging and ECoG recording: The PtNP/graphene electrode array was placed on the cortex centered at 2.2 mm posterior and 2.1 mm lateral relative to bregma, k) Two‐photon microscope was focused on the depth of 250 µm from the cortical surface to detect cell bodies, at the same location with 16× magnification. The deposition time for Electrodes A, B, C, and D is 10, 50, 5, and 5 s, respectively. l) Multiple cells can be imaged, Cells 1 and 2 are directly under the PtNP/graphene electrode, and Cells 3 and 4 are outside. The mean fluorescence change has a region of interest, the same as the electrode^[^ ^270^ ^]^ © 2018 WILEY‐VCH Verlag GmbH & Co. KGaA, Weinheim.

An ultrasensitive, broadband TUT with high optical transparency was developed using a silicon dioxide‐epoxy composite material for matching and backing layers, which have a acoustic impedances of 7.5 and 4–6 MRayl, respectively (**Figure** **6**a–c).^[^ ^251^ ^]^ Figure 6a represents an optical image of the TUT with an excellent optical transparency of >80% and a 63% bandwidth at a single resonance frequency, similar to a traditional opaque ultrasound transducers. Optical transmittance of the developed TUT is relatively low in the visible region but has similar transmittances in the NIR range. A comparison of ultrasound plus‐echo responses between conventional and developed TUTs showed similar performance. The high‐performance TUT maximizes acoustic power and transfer efficiency with minimal ringdowns, enabling high‐definition dual‐modal ultrasound with high contrast and photoacoustic imaging. In vivo imaging of a human palm with TUT revealed blood vessels, demonstrating high definition images with depth‐to‐resolution ratios above 500 and 370, respectively. This advancement sets a new goals for TUTs, enhancing sensor fusion prospective.^[^ ^251^ ^]^

A photoacoustic imaging setup featuring a single‐element TUT window, utilizing ITO‐coated lithium niobate (LiNbO_3_) piezoelectric material, has been developed (Figure 6d,e).^[^ ^252^ ^]^ This setup was demonstrated for applications in endoscopy and microscopy PAI. The study investigated transparent piezoelectric material LiNbO_3_ and lead magnesium niobate‐lead titanate (PMN‐PT) with various matching layer designs, characterized using pulse‐echo and impedance. Its PAI performance was assessed using photoacoustic A‐line signals from light‐absorbing targets. The developed TUTs are easy to fabricate, affordable, and can be easily incorporated into diverse PAI geometries, including endoscopy, microscopy, and computed tomography systems, making them suitable for high‐throughput imaging applications.^[^ ^252^ ^]^

An ultraviolet‐transparent ultrasound transducer (UV‐TUT) (Figure 6f) has been developed using PVDF film and silver nanowires.^[^ ^253^ ^]^ The light transmittance of lithium niobate (LNO), PMN‐PT, and PVDF film was compared, revealing PVDF as the highest transmittance of 96.2% per unit thickness, followed by LNO with 68.9% of and PMN‐PT with 59.9% of. Consequently, PVDF was selected for the UV‐TUT. AgNWs were chosen for their transparency in the UV region. Epo‐Tek 305 was used for bonding layers due to its high UV transmittance. The UV‐TUT demonstrated a transmission efficiency of 61.1% at 266 nm and exhibited four times higher acoustic performance compared to ring‐shaped US transducers. High‐resolution reflection‐mode ultraviolet photoacoustic microscopy (UV‐PAM) was achieved, enhancing numerical aperture and spatial resolution. The UV‐TUT‐PAM's high‐resolution capabilities were showcased by examining the sagittal section of the mouse brain, revealing detailed brain tissue structures. The system's performance was compared with conventional reflection‐mode UV‐PAM, showing superior imaging of brain tissue and cancerous animal tissues. This label‐free reflection‐mode UV‐PAM system holds promise for intraoperative photoacoustic histopathology.^[^ ^253^ ^]^

### 3.3 Optomechanical Sensing

Optomechanical sensing relies on the interaction between mechanical and optical resonances. It has garnered significant interest in sensor applications because its ultra‐sensitivity, compact size,, low detection limit, and has no effect of electromagnetic interference. This type of sensing exploits various mechanisms, such as x changes in optical path length due to mechanical movements, variation in evanescent field coupling, and microscopic photon‐phonon coupling induced by photo‐elastic effects and moving interfaces. Various con configurations of optomechanical sensors have been developed based on these principles,^[^ ^120^ ^]^ such as freestanding microdisk cavities, photonic crystal waveguides on cantilevers, and Fabry–Perot cavities. A high‐Q coherent optomechanical oscillator has been reported utilizing an optical spring effect with significantly enhanced sensing resolution, enabling the detection of single bovine serum albumin (BSA) proteins (66 kDa) with signal‐to‐noise ratio of 16.8.^[^ ^254^ ^]^ The optical spring sensing principle was explained using a ≈100 µm silica microsphere. The device demonstrates an intrinsic optical as high as 4.8 × 10^6^ in an aqueous environment at a wavelength ≈974 nm., With this optical wave inside a strong radiation pressure is produced inside the microsphere that effectively drives the radial breathing mechanical motion of the microsphere (Figure 6g). Injecting an optical power of 3.0 mW into the cavity elevated the mechanical mode above its limit, even within the aqueous environment, resulting in coherent optomechanical oscillations at 262 kHz. This has an exceptional narrow mechanical linewidth of 0.1 Hz, leading to an efficient mechanical Q factor of 2.6 × 10^6^ has been observed.^[^ ^120^ ^]^ This unique optical spring sensing approach offers a novel method for single biomolecule detection and holds promise for a wide range of physical sensing applications by detecting optical cavity resonance shifts. The ultrahigh sensitivity has been demonstrated on the silica nanobead strongly suggests the ability to sense a single protein molecule, BSA solution prepared in Dulbecco's phosphate‐buffered saline around the microsphere sensor, with the increasing concentration ranging from 0 to 100 nm. Figure 6g spectrogram indicates stable optomechanical oscillations without any BSA molecules. Additionally, the histogram of all 118 steps demonstrates a clear difference in the baseline signal and BSA signal detected in experiments. This demonstrates the ability to sense a single BSA molecule with a molecular weight of 66 kDa.^[^ ^254^ ^]^ A sensing platform combining quantum engineering with single‐molecule biophysics has been developed on the surface of a bulk diamond crystal for immobilization of individual proteins and DNA molecules that host coherent nitrogen‐vacancy qubit.^[^ ^223^ ^]^ Diamond‐based sensing shows excellent surface morphology and surface coverage while relying on minimizing the thickness of any functionalization layer. Step‐by‐step functionalization of pristine diamond surface was confirmed by atomic force microscopy, and further a 2 nm thick Al_2_O_3_ was deposited using thermal atomic layer deposition creating a uniform, excellent surface morphology on an oxygen‐terminated diamond surface. To confirm the presence of aluminum (especially the Al2p signal) after each surface treatment step X‐ray photoelectron spectroscopy (XPS) analysis was performed, demonstrating a stable Al_2_O_3_ layer during the processing. Angle‐resolved XPS further confirmed the thickness of the deposited Al_2_O_3_ 2.0 ± 0.1 nm and PEG layer 1.2 ± 0.2 nm. This thin (sub‐5 nm) substrate allows precise control over biomolecule adsorption density during functionalization with near‐surface qubit coherence approaching 100 µs. The developed architecture shows highly stability, and biocompatibility representing its potential in different biomedical applications.^[^ ^255^ ^]^

Inspired by the chameleon's mechanochromic mechanism and the spider slit organ microcracked structure, a flexible, ultra‐sensitive dual‐signal optical/electrical skin (OE‐skin) has been designed.^[^ ^256^ ^]^ The OE‐skin has an interactive feedback response to complex stimuli in human‐readable structural colors. It consists of an ionic electrode integrated with a PDMS dielectric layer, a chromotropic layer consisting photonic crystals of ferroferric oxide‐carbon magnetic arrays (Fe_3_O_4_@C), and a hybrid conductive microcracked carbon nanotube/MXene layer. The transparency of polyacrylamide (PAM) and PDMS increases to 85% with gelatin/PAM assembly, suitable for the transmittance of structural color. The SEM images of the ultrathin chromotropic film containing Fe_3_O_4_@C magnetic particles in the gelatin/PAM matrix show a nonclose‐packed arrangement of orthorhombic and triclinic crystal lattices due to their sufficiently large center‐to‐center distance arrangement. In TEM images the orthorhombic photonic crystals with a center‐to‐center distance of 530 nm along the magnetic field direction and a spacing of 610 nm in the orthogonal direction, with corresponding Fe and O elemental maps has been observed. MXene nanosheets were prepared by selectively etching the phase precursor (Ti_3_AlC_2_) in an acidic solution. and obtained a loosely stacked ultrathin multilayer nanosheets of 1.8 nm thickness and ≈3.6 µm lateral size. The stretching of the OE‐skin results in microcracks on the brittle CNT/MXene thin film, which opens, widens, and closes during continuous stretching and releasing of the film leading to a drastic change in resistance and ultrahigh strain sensitivity. The electrode‐PDMS dielectric layers act as a capacitive pressure sensor, the mechanochromic photonic crystals in the gelatin/polyacrylamide stretchable hydrogel film sense strain and pressure stimuli with bright color converting output in the visible spectrum. The conductive microcracked layer has an ultrasensitive strain sensing capability with a gauge factor of 191.8. The responsive behavior of the chromotropic OE‐skin was studied by measuring reflected wavelengths and capacitance signals at different compressive strains. The initial dark red color shifted significantly to orange, yellow, green, blue, and violet as the compressive strain increased to 60%. When applying normal pressure, the thickness of the chromotropic layer decreased, reducing the center‐to‐center distance of the embedded photonic crystals. A decrease in the wavelength of structural colors has been observed with increasing strain, with a linear slope of 4.14 nm %^−1^. Compressing the OE‐skin to 40% strain caused the green letters “UST” to appear, simulating a complex pressure source. On increasing the strain to 60%, the digital number “123” appeared with a blueshifted violet structural color demonstrating that the chromotropic OE‐skin can visually identify the shape and location of complex, with spatially distributed pressures. The developed multilayered OE‐skin has a fast response time and an accurate capacitive pressure sensing ability with a detection limit of 75 Pa. It has a long‐term stability over 5000 cycles along with high‐resolution spatial color on deformation. These findings will help in exploring the potential of the OE‐skins as multifunctional sensing devices.^[^ ^256^ ^]^

A novel skin‐inspired multimodal sensor for real time monitoring of mechanical thermal changes has been studied using environment‐tolerant hydrogel–elastomer hybrid with a sandwich structure.^[^ ^257^ ^]^ An ionic hydrogel has been synthesized with a semi‐interpenetrating network using sodium carboxymethyl cellulose as a nanofiller, PAM as a polymer matrix, and lithium chloride as an ionic transport conductor. PDMS elastomer has been used to encapsulate the hydrogel providing it mechanical strength, water resistance properties and long term water retention (>98%). Addition of silane improves the interfacial bonding and integrity of both hydrogel (S‐PAM) and elastomer (S‐PDMS). The hybrid shows high biocompatibilty along with improved transmittance (≈91.2%), resistance and fatigue. These hybrids have multifunctional sensing capabilities such as real‐time temperature response (temperature coefficient of resistance, ≈−1.1% °C^−1^), broad‐range strain sensing (gauge factor ≈3.8), wide temperature sensing range (from −20 to 60 °C), with underwater information transmission. The S‐PAM hydrogel shows outstanding flexibility at −20 °C and has no characteristic peak in the range of −80 and 20 °C during differential scanning calorimetry. The carboxymethyl cellulose based hydrogel exhibited a denser porous structure with improved mechanical properties. The hybrid hydrogel‐elastomer shows high transparency with the transmittance of 91.2% in the visible range. Notably, the developed material as dual‐parameter sensing capability can efficiently distinguish the overlapping signals of temperature and strain enabling the detection of magnitude and spatial distribution of force and temperature. Due to the excellent flexibility, conductivity, wide sensing range, with good electrical stability the sensor has been used as wearable sensor for monitoring human motion. The results from continuous human motion demonstrate the long‐term durability and repeatability of the sensor under continuous large deformation. The signals obtained from small motions and its repeatability during repeated motions suggest shows its potential to detect swallowing motion in patients.^[^ ^257^ ^]^

### 3.4 Quantum Sensing

Quantum sensing utilizes quantum phenomena, systems, or properties to measure physical quantities.^[^ ^258^ ^]^ The atomic scale and coherence properties of quantum sensors enable exceptional spatial resolution and sensitivity. These sensors leverage quantum effects to achieve improved sensitivity, with some being as small as a single atom, offering unmatched spatial resolution. These capabilities hold promise for significant advancements in biomedical applications.^[^ ^259^, ^260^ ^]^

There are different quantum sensors proposed for biomagnetic sensing, such as spin‐exchange relaxation free (SERF) atomic magnetometers, superconducting quantum interference device (SQUID) magnetometers, and nitrogen‐vacancy (NV)‐diamond magnetometers. SQUID magnetometeris widely used in detection of biomagnetic signal from human organs, reaching a sensitivity of up to 1 fT Hz^−1/2^.

A solid‐state sensor for magnetic field imaging, based on an integrated OLED has been demonstrated, which provides a robust mapping of the magnetic field utilizing spatially resolved magnetic resonance.^[^ ^224^ ^]^ A sub‐micron magnetic field mapping with a high field sensitivity of ≈160 µT Hz^−1/2^ has been achieved while considering monolithic OLED as an array of individual virtual sensors. The study introduces an OLED‐based chip‐scale, laser‐free magnetic field sensor with commercially viable, manufacturable technology for mapping the magnetic field. The integrated device can sensing the magnetic field using electrically detected magnetic resonance (EDMR) providing optically accessible magnetic field mapping using optically detective magnetic resonance (ODMR). An optical microscope has been used to image the device onto an sCMOS camera for measuring the ODMR,. Although the signal‐to‐noise ratio was low after binning, the spectrum could be fitted using a double Gaussian function. Increasing the binning size, decreased the spatial resolution but enhanced measurement sensitivity. The solid‐state device, combining a π‐conjugated‐polymer‐based OLED and a microwave resonator on the same substrate, measures magnetic fields both electrically (via EDMR) and optically (via ODMR). This architecture enables rapid magnetic mapping without the need of point‐to‐point scanning, presenting potential applications in imaging and quantum magnetic sensing. The underlying mechanism of EDMR and ODMR in OLEDs is established on the spin‐dependent recombination and dissociation dynamics of charge‐carrier pairs in the emitting layer, and an emitting polymer a commercial super yellow poly(phenylene‐vinylene) (PPV) copolymer (SY‐PPV).^[^ ^224^ ^]^

NV‐diamond magnetometers, a rising potential technology for imaging magnetotactic bacteria and magnetically tagged cells with single‐cell resolution, exhibit unique properties such as solid‐state aspect, nontoxicity, and room temperature compatibility, though they have relatively low sensitivity.^[^ ^261^, ^262^, ^263^ ^]^ There are two implementations: single or few NV centers in diamond nanoparticles enable nanoscale magnetic‐field detection with sensitivity up to µT Hz^−1/2^ and dynamic sensitivity up to nT Hz^−1/2^.^[^ ^264^, ^265^ ^]^ Second, NV has a high‐density nitrogen‐vacancy centers in bulk diamond offer improved sensitivity over a wide frequency range.^[^ ^266^ ^]^ NV centers in diamond have been utilized for quantitative detection and wide‐field imaging of biological samples labeled with magnetic nanoparticles (MNPs) ples, offering micrometer‐scale resolution along with the view in millimeter range.^[^ ^259^, ^267^ ^]^ NV‐based magnetic microscopy has resolved and quantitatively characterized chains of MNPs (magnetosomes) in magnetotactic bacteria.^[^ ^225^ ^]^ Figure 6h illustrates the quantum diamond microscope, which utilizes a dense layer of fluorescent quantum sensors based on NV color centers located near the surface of a diamond chip where the sample of interest is positioned. The microwaves has been used to examine the electronic spins of the NV centers and are optically initiated and read out provides the spatially resolved maps of local magnetic fields. This method has been used to detect cancer biomarkers in SKBR3 cancer cells labeled with HER2‐specific MNPs, distinguishing between healthy and cancer cells. Aa solution containing both magnetically labeled and unlabeled cancer cells was drop cast on the diamond surface, and bright field, fluorescence, and magnetic images were acquired, showing a good signal‐to‐noise ratio. The unlabeled cells were eliminated in less than 1 minute of magnetic signal acquisition. This technique is commercially available for diagnosing biomarkers in human blood and other samples through Quantum Diamond Technologies Inc. (QDTI).^[^ ^225^ ^]^ NV‐diamond magnetic microscopy has also been used to study malarial haemozoin nanocrystals, demonstrating their paramagnetic nature and measuring a magnetic susceptibility of 3.4 × 10^−4^.^[^ ^230^ ^]^ Bringing very small biological samples close to NV centers in a macroscopic diamond chip can be challenging: however, nanodiamonds having NV centers offer a viable alternative. Nanodiamonds can be introduced into cells, tissues, and other biological samples, and surface functionalization facilitates targeting biological molecules such as proteins of interest.

NV centers in nanodiamonds are also used for nanoscale detection of free radicals in biological samples, which are associated with neurological disorders and cardiovascular diseases.^[^ ^268^ ^]^ Free radicals has a crucial role in the immune system, and their sensitive detection with subcellular spatial resolution is vital for understanding biological processes. T1 relaxometry using NV centers in nanodiamonds allows for this precise detection and has been employed to study free radicals in single mitochondria and human dendritic cells.^[^ ^259^ ^]^

A study by Shi et al.^[^ ^269^ ^]^ demonstrates a design for pressure sensors with ultrahigh sensitivity and sensing density based on the Fowler–Nordheim (F–N) tunneling effect.^[^ ^269^ ^]^ The incorporation of urchin‐like hollow carbon spheres (UHCSs) at a low concentration contributes to the ultrahigh sensitivity of the pressure sensor through the F–N tunneling effect. The F–N effect is a quantum tunneling phenomenon that allows electrons to pass through a dielectric without damaging its structure when subjected to an suitable external electric field. This effect is highly sensitive to changes in distance, making it ideal for pressure‐sensing applications. The UHCSs are synthesized with long, sharp spines that enable concentrating the strong local electric field crucial for the F–N tunneling effect to occur. Upon compression, the UHCSs embedded in the sensor do not physically contact each other due to the PDMS medium and low filler loading. However, the pressure changes the distance between the spines of adjacent UHCSs, leading to the change in the resistance of UHCS‐PDMS and affecting the tunneling current. This changes the current of the local electric field strength and the tunneling distance, which are both influenced by the applied pressure. The low concentration of UHCSs (<1.5 wt.%) ensures that the sensor remains in the F–N tunneling region without reaching the percolation threshold, where the sensor would exhibit different electrical behavior. This concentration is optimized to achieve a hyper‐exponential increase in current density with pressure, leading to the ultrahigh sensitivity observed in the sensor. The fabrication of an ultra‐small (5 µm × 5 µm) pressure sensor device using UHCS has been done in the following steps. First, the synthesis of UHCSs were done using a seeded swelling polymerization method. Polystyrene (PS) seed colloidal spheres were dispersed in an aqueous of aniline solution. This solution was polymerized using Fe(NO_3_)_3_, resulting in the formation of spiky particles having polyaniline spikes on the surface of the PS surface. These particles were then carbonized to remove PS core and improve conductivity. These particles were then dispersed in PDMS prepolymer at a concentration of 1.43%, then spin‐coated onto a hydrophobic silicon wafer and finally cured for 3 h at 80 °C to form a thin film. The device fabrication was done by sandwiching the thin film between two metallic lines. This involves patterning the electrodes on the substrate and then encapsulating the sensing material between them to create a resistive‐type pressure sensor. The sensor's performance is evaluated in terms of its sensitivity, response time, and stability under various conditions, including different pressures and temperatures. This sensor shows an ultrahigh sensitivity of 260.3 kPa at 1 Pa and demonstrates a high sensing density of 400 cm^−2^, along with high transparency and temperature noninterference. Furthermore, the scalable spin‐coating method enables the sensor to be applied as an ultrahigh sensitivity flexible pressure sensor on different surfaces and in in vivo environments. The study successfully demonstrates the fabrication an F–N effect‐based ultra‐sensitivity pressure sensor by spin‐coating a very low concentration of UHCSs, <1.5 wt.%) dispersed in PDMS, staying below the percolation threshold. The thin film achieves 87% transmittance across wavelengths from 400 to 1500 nm, due to the low loading of the filler and small particle size of the UHCSs. A compact (5 × 5 µm) pressure sensor device was created by placing the thin film between two metallic lines (Figure 6d). This sensors demonstrate an ultrahigh sensitivity of 260.3 kPa^−1^ at 1 Pa, outperforming previously reported sensors, and demonstrate conduction in vertical‐direction and insulation in horizontal‐direction under pressure, achieving a theoretical sensing density value of 2 718 557 per cm^2^. At 1.43% concentration of UHCS and electrode thickness of 35 nm the calculations estimate the minimum sensing area of 31.7 µm and minimum pitch size to be 435 nm, respectively,, assuming a reliable readout probability of >97%. Two experiments were conducted to verify these theoretical values; first, by fabricating tiny, patterned electrodes with a width of 5 µm on a wafer and positioning them perpendicularly with a thin film in between to create a sensing system. The pressure sensor maintains high transparency, elasticity, skin compatibility, and excellent processability. Its hollow structure enables to resist the temperature interference, making it suitable for use on surfaces such as human skin, monitors, cameras, and displays, and for extensive data collection in big data technology.^[^ ^269^ ^]^

A study by Lu et al.^[^ ^270^ ^]^ demonstrates that graphene microelectrodes showing high impedance is fundamentally constrained by quantum capacitance.^[^ ^270^ ^]^ This limitation was overcome by establishing a parallel conduction path with platinum nanoparticles, resulting in a 100‐fold decrease in graphene electrode impedance while preserving the high optical transparency essential for deep two‐photon microscopy. Figure 6i shows graphene microelectrode arrays with electrode size of 100 µm and spacing of 400 µm, featuring chromium 10 nm and gold 100 nm deposited onto a PET substrate to form metal wires and contact pads. The impedance measured at 1 KHz showed an average impedance of 872.9 KΩ. The equivalent circuit model used to calculate the impedance of the graphene microelectrode includes *R*_s_ (solution resistance), CPE (constant phase element demonstrating Helmholtz double‐layer capacitance), WB (bounded Warburg element simulating the diffusion process), and *R*_ct_ (charge‐transfer resistance representing Faradaic reactions), with the quantum capacitance of graphene in series with the CPE. Using a transgenic mouse model for in vivo, the system achieved precise electrical recording of cortical activity and imaging calcium signals at various cortical depths beneath the transparent microelectrodes. This multimodal analysis of Calcium Ions (Ca^2+^) spikes and cortical surface potentials provides insights into cellular dynamics and brain‐scale neural activity. Bregma is used as a reference to place the electrode as it is a standard practice in neuroscience experiments, particularly in rodent studies.^[^ ^271^ ^]^ It provides consistency and reproducibility by ensuring that the electrodes are placed in consistent locations across different animals, which is crucial for comparing results between different experimental groups. Being an intersection point, it helps in targeting specific regions of the brain. The brain is organized into distinct regions, each responsible for a specific role. These coordinates are determined by positioning the animal's head in a stereotaxic apparatus and identifying bregma as the zero point for the 3D coordinate system (anterior‐posterior, medial‐lateral, and dorsal‐ventral). It helps to confirm the placement of the electrodes relative to the target regions post‐surgery. The use of bregma for referencing has several specific implications such as in spatial resolution, and functional mapping to relate neural activity to behavior or cognitive process and helps in network analysis revealing how different regions of the brain interact and contribute to complex information processing tasks.^[^ ^271^ ^]^ Overall, it helps in better understanding of neural signaling and improving treatment to neurological disorders.^[^ ^272^, ^273^ ^]^ Figure 6j shows the placement of the PtNPs/graphene electrode array on the cortex, centered 2.2 mm posterior and 2.1 mm lateral to bregma, and Ca^2+^ response is clearly visible from cell bodies at 250 µm (Figure 6k), electrodes labelled as A, B, C and D in Figure 6k represents PtNPs deposition time of 10, 50, 5, and 5 s, respectively. Figure 6l represents the maximum intensity projection of image stacks recorded over 5 min at electrode B, clearly displaying the cell bodies with high magnification. For quantification of the cells (F ‐F0)/F0 was used, here F represents the intensity of the fluorescence from the pixels under each electrode, F0 is the 8th percentile of the distribution of the intensity for the complete recording session.^[^ ^270^, ^274^, ^275^ ^]^ The (F – F0)/F0 formula is used in fluorescence imaging to quantify the changes in fluorescence intensity. This formula calculates the relative changes in the fluorescence intensity as a proportion of the baseline fluorescence. F represents the mean fluorescence intensity of the pixels within the region of interest (ROI) at a certain time point expressing fluorescent calcium indicator. F0 is the baseline fluorescence intensity within the ROI, measured when there is no significant neural activity or calcium influx into the cells. (F – F0) calculates the difference between the fluorescence intensity of a certain time point and baseline intensity. It reflects the change in fluorescence intensity due to neural activity associated with an increase in intracellular calcium concentration.^[^ ^270^, ^274^, ^275^ ^]^ To further investigate the relationship between surface potentials and Ca^2+^ signals, multimodal data consisting of Ca^2+^ spikes and µECoG potentials were analyzed. The power of ECoG oscillations preceding Ca^2+^ peaks at both depths shows the consistent trend across a wide frequency range, from δ to high‐γ band. However, at 50 µm, the power ratios associated with Ca^2+^ signals at were higher in the low‐frequency bands (δ and θ) and lower in the high‐frequency bands (γ and high‐γ) compared to those at 250 µm. This indicates that synaptic activity from the neuropil mainly contributes to slower ECoG oscillations, while spiking activity from cell bodies in deeper layers are responsible for higher frequency bands such as γ and high‐γ.^[^ ^270^ ^]^

## 4 Challenges and Future Outlook

Transparent electrodes, crucial in advancing multimodal sensing technologies, have garnered significant attention in bioelectronics due to their exceptional optical properties, high conductivity, and mechanical flexibility. These electrodes, developed from various materials, including carbon‐based compounds, conductive polymers, metal‐based elements, and composite/hybrid materials, are increasingly utilized in diverse healthcare applications, including robotics, epidermal electronics and environmental monitoring. This review underscores the latest advancements in novel materials, their intricate structures, sensing mechanisms, and their integrative combinations for enhancing multimodal sensors. However, the journey toward optimizing these materials in terms of processability and performance is marked by numerous challenges. Realizing the full potential of these materials and technology requires addressing these challenges.

A key challenge in the realm of multimodal sensing is the enhancement of conductivity and mechanical stability in stretchable and transparent electrodes. Each material, while promising, exhibits inherent limitations such as limited conductivity or mechanical instability. The development of hybrid materials by combining the properties of different materials like graphene, carbon nanotubes, silver nanowires, and conductive polymers can lead to the development of more robust and conductive electrodes.^[^ ^276^, ^277^ ^]^ Emphasizing hybridizing various conducting materials, including 1D and 2D structures, can help improve mechanical durability while ensuring consistent electrical conductivity.^[^ ^278^, ^279^ ^]^ These hybrid materials with improved electrical and optical properties can exceed the performance of the traditional ITO electrodes.

Along the same lines, another crucial area that needs improvement is the mechanical deformability of TEs without compromising their electrical conductivity and light transparency. A systematic study is needed to establish mechanisms for TEs resistance to bending and stretching deformations. Additionally, creating fiber‐shaped TEs is vital for breathable wearable electronics, as traditional film‐based electrodes lack air permeability, potentially causing skin issues. To enhance the mechanical stability and durability, the use of flexible substrates such as PDMS, PEDOT:PSS with PVDF can also be proposed. Applying a protective coating to protect the electrodes from different factors such as mechanical wear and tear, and environmental factors can extend their durability.^[^ ^280^ ^]^

In the broader context of multimodal sensors, scalability in device fabrication is another significant challenge, particularly for applications in robotics or prosthetics. The complexity of fabrication processes and the high material costs call for exploring economical manufacturing techniques and optimized designs. In addition, developing integrated, transparent, stretchable platform systems is crucial, particularly for applications like electronic skin, human activity monitoring, and personal healthcare. These systems should include sensors with the microcontroller units, power generators, and data transmission modules, enhancing comprehensive biological and environmental monitoring capability. Toward this aim, developing economically scalable fabrication techniques that ensure uniformity, stretchability, and integration with optoelectrical components without compromising performance can be used in future research. For example, techniques like chemical vapor deposition, inkjet and screen printing having optimized design and reducing material cost and complexity can help in scaling up.

An efficient integration of electrical and optical signals to minimize losses and interference can be done by optimizing electrode design, integrating multilayer structures can enhance the overall performance of the device. A balance between high electrical conductivity and effective charge transfer is essential for high‐efficiency flexible devices. While metallic electrodes offer advantages, alternatives like PEDOT: PSS and carbon electrodes often require additional doping to enhance performance. The pursuit of flexible electrodes that integrate high electrical conductivity with light transmittance continues, aiming to bridge the gap between flexible and rigid devices for efficient optoelectronic applications. While maintaining the high efficiency and performance of optoelectrical devices reducing power consumption is also a challenge. Integration of low‐power electronics with optimized circuits can help minimize power consumption. Adding energy harvesting technologies to supplement power needs can also enhance device power usage.

Cost is a primary consideration for the future commercialization of transparent electrodes. Therefore, the expenses associated with transparent and flexible electrodes, which primarily include raw material and manufacturing costs, must be carefully evaluated. The most commonly used electrodes in transparent flexible electronics are ITO and metal films. However, the limited availability of indium and the high price of gold and silver increases the costs of flexible electrodes. In contrast, due to the abundance of carbon, carbon‐based materials, such as transparent CNTs and graphene, opaque carbon materials, make attractive options for achieving low‐cost flexible electrodes. Additionally, using inexpensive copper film instead of gold and silver films can significantly reduce fabrication costs. Beyond material costs, the need for high vacuum conditions and high‐temperature annealing during the fabrication of ITO and metal films further raises the overall manufacturing expenses. Producing metal mesh electrodes are typically involves expensive or time‐consuming methods such as photolithography and nano‐imprinting. Moreover, the c transfer of complex and lamination procedures required for fabricating CNT and graphene electrodes are not compatible with continuous roll‐to‐roll manufacturing. Solution‐processable electrodes, like PEDOT, carbon pastes, and AgNWs, show promise for cost effective and large‐scale production. New fabrication techniques must be developed to enable scalable roll‐to‐roll manufacturing of flexible electrodes for commercialization. An analysis of the cost‐effectiveness of different transparent electrode materials is provided in Table S5 (Supporting Information). This table compares the cost factors and economic benefits of various transparent electrode materials, highlighting the potential for large‐scale production and commercialization.

Another technical challenge associated with transparent electrodes in commercial applications is their large‐scale production. Developing cost‐effective, large‐scale production methods for high‐quality CNTs and graphene is essential. For example, stability in chemical doping of CNT and graphene‐based TEs is crucial to address device instability despite their comparable optoelectrical performance to traditional materials.^[^ ^281^ ^]^ Producing large‐area graphene‐based TEs that effectively balance high electrical conductivity with transparency remains a critical area of research.

The biocompatibility of the transparent electrode‐based device is also a concern for medical applications and environmental safety. Selecting nontoxic and biocompatible materials such as zinc oxide, and silver nanowires has shown promise toward biomedical applications.^[^ ^282^ ^]^ In addition to this adhering to the regulatory standards for fabricated devices in medical use and environmental safety can be prospective during fabrication.

Finally, defining standardized criteria for evaluating the flexibility and stretchability of TEs is imperative. The diversity in testing methods currently poses a challenge in selecting TEs with superior mechanical properties. Addressing these issues will be pivotal in advancing the production process and meeting the demands of wearable electronics. A comparison of the properties of transparent and conventional electrodes is summarized in Table S6 (Supporting Information).

While the path ahead is challenging, the relentless pursuit of innovation in this field is set to revolutionize the landscape of electronics. The ongoing research endeavors are poised to catalyze advancements in the development of stretchable and transparent electronic devices, heralding a new era of increased functionality and broader applications in the near future, particularly in enhancing the capabilities of multimodal sensing technologies.

## 5 Conclusion

This review highlights the significant role of transparent electrodes in advancing multimodal sensing technologies, noting their increasing utilization in sectors such as healthcare, robotics, and epidermal electronics. Despite the progress in developing these electrodes from various materials like carbon‐based compounds, conductive polymers, and metal‐based elements, numerous challenges persist. Enhancing conductivity and mechanical stability in stretchable and transparent electrodes remains a key focus, with future research poised to explore the hybridization of conducting materials and structural engineering approaches. The scalability of device fabrication, especially for robotics and personal healthcare applications, demands the development of cost‐effective manufacturing techniques and integrated systems. Technical challenges in commercial applications include the need for large‐scale production of high‐quality materials like carbon nanotubes and graphene and the quest for flexible electrodes that effectively combine electrical conductivity with light transmittance. Addressing these challenges is crucial for advancing the production process and meeting the demands of wearable electronics. The review concludes with an optimistic outlook on the relentless pursuit of innovation in transparent electrodes, anticipating a revolution in the field that promises enhanced functionality and broader applications, particularly in multimodal sensing technologies.

## Conflict of Interest

The authors declare no conflict of interest.

## Supporting information

Supporting Information

## References

[1] D. S. Hecht , L. Hu , G. Irvin , Adv. Mater. 2011, 23, 1482. 21322065 10.1002/adma.201003188

[2] H. Wu , D. Kong , Z. Ruan , P. C. Hsu , S. Wang , Z. Yu , T. J. Carney , L. Hu , S. Fan , Y. Cui , Nat. Nanotechnol. 2013, 8, 421. 23685985 10.1038/nnano.2013.84

[3] K. Ellmer , Nat. Photonics 2012, 6, 809.

[4] J. Liang , L. Li , X. Niu , Z. Yu , Q. Pei , Nat. Photonics 2013, 7, 817.

[5] J. A. Rogers , T. Someya , Y. Huang , Science 2010, 327, 1603. 20339064 10.1126/science.1182383

[6] D. Li , W. Y. Lai , Y. Z. Zhang , W. Huang , Adv. Mater. 2018, 30, 04738. 10.1002/adma.201704738 29319214

[7] E. Yang , S. Kang , S. Jeong , K. Shin , J.‐S. Wi , J. S. Park , S. Lee , C.‐H. Chung , Electron. Mater. Lett. 2024, 20, 254.

[8] Q. Fan , J. Miao , X. Liu , X. Zuo , W. Zhang , M. Tian , S. Zhu , L. Qu , X. Zhang , Nano Lett. 2022, 22, 740. 35019663 10.1021/acs.nanolett.1c04185

[9] X. Meng , X. Hu , X. Yang , J. Yin , Q. Wang , L. Huang , Z. Yu , T. Hu , L. Tan , W. Zhou , Y. Chen , ACS Appl. Mater. Interfaces 2018, 10, 8917. 29457446 10.1021/acsami.8b00093

[10] Y. Chen , J. Wan , G. Xu , X. Wu , X. Li , Y. Shen , F. Yang , X. Ou , Y. Li , Y. Li , Sci. China Chem. 2022, 65, 1164.

[11] L. Lu , ACS Appl. Bio Mater. 2023, 6, 1701. 10.1021/acsabm.3c00131 37076978

[12] S. Zhou , H. Zhang , X. Peng , H. Liu , H. Li , Y. Xiong , W. Li , P.‐A. Yang , L. Ye , C. Kong , Adv. Photon. Res. 2022, 3, 2270037.

[13] M. Kim , H.‐K. Um , H. Choi , J. S. Lee , J. Kim , K. Kim , E. Noh , M. Han , H. W. Lee , W. I. Choi , S. H. Lee , J.‐R. Lee , B. H. Lee , Adv. Electron. Mater. 2023, 9, 2300075.

[14] A. Takemoto , T. Araki , T. Uemura , Y. Noda , S. Yoshimoto , S. Izumi , S. Tsuruta , T. Sekitani , Adv. Intell. Syst. 2020, 2, 2000093.

[15] B. Han , Y. Huang , R. Li , Q. Peng , J. Luo , K. Pei , A. Herczynski , K. Kempa , Z. Ren , J. Gao , Nat. Commun. 2014, 5, 6674. 10.1038/ncomms6674 25430671

[16] A. Zhuang , X. Huang , S. Fan , X. Yao , B. Zhu , Y. Zhang , ACS Appl. Mater. Interfaces 2022, 14, 123. 34935351 10.1021/acsami.1c16855

[17] B. Massih , A. Veh , M. Schenke , S. Mungwa , B. Seeger , B. T. Selvaraj , S. Chandran , P. Reinhardt , J. Sterneckert , A. Hermann , M. Sendtner , P. Lüningschrör , Front. Cell Develop. Biol. 2023, 11, 996952. 10.3389/fcell.2023.996952 PMC9973451 36866276

[18] Y. Shen , Y. Yuan , X. Liu , T. Tang , Y. Yalikun , Y. Tanaka , IEEE Sens. J. 2023, 23, 1795.

[19] X. Ma , S. Ahadian , S. Liu , J. Zhang , S. Liu , T. Cao , W. Lin , D. Wu , N. R. de Barros , M. R. Zare , S. E. Diltemiz , V. Jucaud , Y. Zhu , S. Zhang , E. Banton , Y. Gu , K. Nan , S. Xu , M. R. Dokmeci , A. Khademhosseini , Adv. Intell. Syst. 2021, 3, 2000263.

[20] Y. Zhu , S. Li , J. Li , N. Falcone , Q. Cui , S. Shah , M. C. Hartel , N. Yu , P. Young , N. R. de Barros , Z. Wu , R. Haghniaz , M. Ermis , C. Wang , H. Kang , J. Lee , S. Karamikamkar , S. Ahadian , V. Jucaud , M. R. Dokmeci , H.‐J. Kim , A. Khademhosseini , Adv. Mater. 2022, 34, 2108389. 10.1002/adma.202108389 PMC9233032 35130584

[21] N. L. Kazanskiy , S. N. Khonina , M. A. Butt , Biosensors (Basel) 2023, 13, 933. 37887126 10.3390/bios13100933 PMC10605521

[22] Z. Fan , J. C. Ho , T. Takahashi , R. Yerushalmi , K. Takei , A. C. Ford , Y. L. Chueh , A. Javey , Adv. Mater. 2009, 21, 3730.

[23] L. Chen , X. Chang , H. Wang , J. Chen , Y. Zhu , Nano Energy 2022, 96, 107077.

[24] J. H. Kim , S. R. Kim , H. J. Kil , Y. C. Kim , J. W. Park , Nano Lett. 2018, 18, 4531. 29923729 10.1021/acs.nanolett.8b01743

[25] D. H. Kim , J. Xiao , J. Song , Y. Huang , J. A. Rogers , Adv. Mater. 2010, 22, 2108. 20564250 10.1002/adma.200902927

[26] J. Perelaer , P. J. Smith , D. Mager , D. Soltman , S. K. Volkman , V. Subramanian , J. G. Korvink , U. S. Schubert , J. Mater. Chem. 2010, 20, 8446.

[27] X. Wang , E. G. Lim , K. Hoettges , P. Song , C 2023, 9, 108.

[28] A. Ali , S. S. Rahimian Koloor , A. H. Alshehri , A. Arockiarajan , J. Mater. Res. Technol. 2023, 24, 6495.

[29] X. Zhang , Z. Li , C. Liu , J. Shan , X. Guo , X. Zhao , J. Ding , H. Yang , ACS Appl. Nano Mater. 2022, 5, 15797.

[30] S. Gupta , R. Datt , A. Mishra , W. C. Tsoi , A. Patra , P. Bober , J. Appl. Polym. Sci. 2022, 139, e52663.

[31] P. Won , J. J. Park , T. Lee , I. Ha , S. Han , M. Choi , J. Lee , S. Hong , K.‐J. Cho , S. H. Ko , Nano Lett. 2019, 19, 6087. 31411037 10.1021/acs.nanolett.9b02014

[32] L. Zhang , T. Song , L. Shi , N. Wen , Z. Wu , C. Sun , D. Jiang , Z. Guo , J. Nanostruct. Chem. 2021, 11, 323. 10.1007/s40097-021-00436-3 PMC8325546 34367531

[33] J. Chen , C. Ye , T. Cang , R. Gao , X. Li , J. Mater. Chem. C 2023, 11, 14930.

[34] H. Lee , Z. Jiang , T. Yokota , K. Fukuda , S. Park , T. Someya , Mater. Sci. Eng. R: Rep. 2021, 146, 100631.

[35] K. Zhang , L. Feng , L. Wang , J. Zhu , H. Zhang , S. Ha , J. Sun , H. Liang , T. Yang , Crystals 2023, 13, 1018.

[36] Y. Li , Highlights Sci., Eng. Technol. 2023, 43, 315.

[37] L. Li , B. Zhang , B. Zou , R. Xie , T. Zhang , S. Li , B. Zheng , J. Wu , J. Weng , W. Zhang , W. Huang , F. Huo , ACS Appl. Mater. Interfaces 2017, 9, 39110. 29068225 10.1021/acsami.7b12298

[38] R. Garg , S. Elmas , T. Nann , M. R. Andersson , Adv. Energy Mater. 2017, 7, 01393.

[39] G. U. Kulkarni , S. Kiruthika , R. Gupta , K. D. M. Rao , Curr. Opin. Chem. Eng. 2015, 8, 60.

[40] Y.‐G. Bi , Y.‐F. Liu , X.‐L. Zhang , D. Yin , W.‐Q. Wang , J. Feng , H.‐B. Sun , Adv. Opt. Mater. 2019, 7, 1800778.

[41] F. Yan , X. Zhang , Y. G. Yu , L. Yu , A. Nagaraja , T. O. Mason , A. Zunger , Nat. Commun. 2015, 6, 7308. 26106063 10.1038/ncomms8308

[42] E. Fortunato , D. Ginley , H. Hosono , D. Paine , MRS Bull. 2007, 32, 29.

[43] Q. Zhang , E. Uchaker , S. L. Candelaria , G. Cao , Chem. Soc. Rev. 2013, 42, 3127. 23455759 10.1039/c3cs00009e

[44] K. Allen , K. Connelly , P. Rutherford , Y. Wu , Energy Build. 2017, 139, 535.

[45] W. Guo , Z. Xu , F. Zhang , S. Xie , H. Xu , X. Y. Liu , Adv. Funct. Mater. 2016, 26, 8855.

[46] A. Stadler , Materials (Basel) 2012, 5, 661. 28817002 10.3390/ma5040661 PMC5448960

[47] H. Hosono , K. Ueda , in Springer Handbook of Electronic and Photonic Materials (Eds.: S. Kasap , P. Capper ), Springer International Publishing, Cham 2017, pp. 1–1.

[48] S.‐C. Her , C.‐F. Chang , J. Appl. Biomater. Funct. Mater. 2017, 15, 170. 28430343 10.5301/jabfm.5000345 PMC6379771

[49] G. Lucarelli , T. M. Brown , Front. Mater. 2019, 6.

[50] S. Khan , E. Stamate , Nanomaterials 2022, 12, 1539. 35564248 10.3390/nano12091539 PMC9104591

[51] Z. Chen , J. Wang , H. Wu , J. Yang , Y. Wang , J. Zhang , Q. Bao , M. Wang , Z. Ma , W. Tress , Z. Tang , Nat. Commun. 2022, 13, 4387. 35902576 10.1038/s41467-022-32010-y PMC9334612

[52] M.‐J. Kim , Materials 2023, 16, 4718.37445032

[53] M. L. Hupfer , A. Gawlik , J. Dellith , J. Plentz , Materials 2023, 16, 3961. 37297095 10.3390/ma16113961 PMC10253794

[54] K. Juraić , P. Dubček , M. Bohač , A. Gajović , S. Bernstorff , M. Čeh , A. Hodzic , D. Gracin , Materials 2022, 15, 4814. 35888281 10.3390/ma15144814 PMC9315605

[55] P. Ledochowitsch , E. Olivero , T. Blanche , M. M. Maharbiz , Ann. Int. Conf. IEEE Eng. Med. Biol. Soc. 2011, 2011, 2937. 10.1109/IEMBS.2011.6090808 22254956

[56] I. J. Gómez , M. Vázquez Sulleiro , D. Mantione , N. Alegret , Polymers (Basel) 2021, 13, 745. 33673680 10.3390/polym13050745 PMC7957790

[57] F. Ardente , F. Mathieux , M. Recchioni , Resour., Conserv. Recycl. 2014, 92, 158.

[58] K. Y. Kwon , B. Sirowatka , A. Weber , W. Li , IEEE Trans. Biomed. Circuits Syst. 2013, 7, 593. 24144668 10.1109/TBCAS.2013.2282318

[59] J. P. Neto , A. Costa , J. Vaz Pinto , A. Marques‐Smith , J. C. Costa , R. Martins , E. Fortunato , A. R. Kampff , P. Barquinha , ACS Appl. Nano Mater. 2021, 4, 5737.

[60] J. Lee , I. Ozden , Y.‐K. Song , A. V. Nurmikko , Nat. Methods 2015, 12, 1157. 26457862 10.1038/nmeth.3620

[61] B. Zhuang , Q. Zhang , K. Zhou , H. Wang , RSC Adv. 2023, 13, 18229. 37333797 10.1039/d3ra01701j PMC10274301

[62] S. Habib , F. Rashid , H. Tahir , I. Liaqat , A. A. Latif , S. Naseem , A. Khalid , N. Haider , U. Hani , R. A. Dawoud , Y. Modafer , A. Bibi , O. A. Jefri , Microorganisms 2023, 11, 1363. 37374866 10.3390/microorganisms11061363 PMC10305289

[63] H. B. Lee , W. Y. Jin , M. M. Ovhal , N. Kumar , J. W. Kang , J. Mater. Chem. C 2019, 7, 1087.

[64] J. Yun , Adv. Funct. Mater. 2017, 27, 06641.

[65] K. Zilberberg , T. Riedl , J. Mater. Chem. A 2016, 4, 14481.

[66] S. Devaraju , A. Kumar Mohanty , D. Won , H. Paik , Mater. Adv. 2023, 4, 1769.

[67] L. Hu , H. Wu , Y. Cui , MRS Bull. 2011, 36, 760.

[68] X. Lu , Y. Zhang , Z. Zheng , Adv. Electron. Mater. 2021, 7, 1121.

[69] R. S. Sennett , G. D. Scott , J. Opt. Soc. Am. 1950, 40, 203.

[70] M. Fahland , P. Karlsson , C. Charton , Thin Solid Films 2001, 392, 334.

[71] C. Ji , D. Liu , C. Zhang , L. J. Guo , Nat. Commun. 2020, 11, 3367. 32632111 10.1038/s41467-020-17107-6 PMC7338390

[72] Y. G. Bi , F. S. Yi , J. H. Ji , C. Ma , W. Q. Wang , J. Feng , H. B. Sun , IEEE Trans. Nanotechnol. 2018, 17, 1077.

[73] S. Schubert , L. Müller‐Meskamp , K. Leo , Adv. Funct. Mater. 2014, 24, 6668.

[74] V. K. Bajpai , P. Pant , C. S. Solanki , Sol. Energy 2017, 155, 62.

[75] G. Xu , L. Shen , C. Cui , S. Wen , R. Xue , W. Chen , H. Chen , J. Zhang , H. Li , Y. Li , Y. Li , Adv. Funct. Mater. 2017, 27, 5908.

[76] E. Jeong , G. Zhao , S. M. Yu , S. G. Lee , J. S. Bae , J. Park , J. Rha , G. H. Lee , J. Yun , Appl. Surf. Sci. 2020, 528, 6989.

[77] C. Hanmandlu , C. C. Liu , C. Y. Chen , K. M. Boopathi , S. H. Wu , M. Singh , A. Mohapatra , H. W. Lin , Y. C. Chang , Y. C. Chang , C. S. Lai , C. W. Chu , ACS Appl. Mater. Interfaces 2018, 10, 17973. 29737157 10.1021/acsami.8b04329

[78] M. Girtan , Sol. Energy Mater. Sol. Cells 2012, 100, 153.

[79] Z. Xue , X. Liu , N. Zhang , H. Chen , X. Zheng , H. Wang , X. Guo , ACS Appl. Mater. Interfaces 2014, 6, 16403. 25148532 10.1021/am504806k

[80] J. F. Salinas , H. L. Yip , C. C. Chueh , C. Z. Li , J. L. Maldonado , A. K. Y. Jen , Adv. Mater. 2012, 24, 6362. 23001960 10.1002/adma.201203099

[81] A. Dhar , T. L. Alford , J. Appl. Phys. 2012, 112, 7662.

[82] X.‐L. Ou , J. Feng , M. Xu , H.‐B. Sun , Opt. Lett. 2017, 42, 1958. 28504769 10.1364/OL.42.001958

[83] D. Y. Kim , Y. C. Han , H. C. Kim , E. G. Jeong , K. C. Choi , Adv. Funct. Mater. 2015, 25, 7145.

[84] Y. G. Bi , J. Feng , J. H. Ji , Y. Chen , Y. S. Liu , Y. F. Li , Y. F. Liu , X. L. Zhang , H. B. Sun , Nanoscale 2016, 8, 10010. 27128168 10.1039/c6nr00599c

[85] S. Jeong , S. Jung , H. Kang , D. Lee , S. B. Choi , S. Kim , B. Park , K. Yu , J. Lee , K. Lee , Adv. Funct. Mater. 2017, 27, 06842.

[86] J. Ham , J. L. Lee , Adv. Energy Mater. 2014, 4, 539.

[87] J. Zou , C. Z. Li , C. Y. Chang , H. L. Yip , A. K. Y. Jen , Adv. Mater. 2014, 26, 3618. 24623553 10.1002/adma.201306212

[88] H. M. Stec , R. A. Hatton , ACS Appl. Mater. Interfaces 2012, 4, 6013. 23127805 10.1021/am3016763

[89] H. Wang , Z. Wang , X. Xu , W. Zhao , D. Wu , M. Muhammad , Y. Liu , C. Chen , B. Liu , Y. Duan , Adv. Opt. Mater. 2020, 8, 1320.

[90] H. W. Bae , S. K. Kim , S. Lee , M. G. Song , R. Lampande , J. H. Kwon , Adv. Electron. Mater. 2019, 5, 0620.

[91] S. Schubert , J. Meiss , L. Müller‐Meskamp , K. Leo , Adv. Energy Mater. 2013, 3, 438.

[92] H. Kang , S. Jung , S. Jeong , G. Kim , K. Lee , Nat. Commun. 2015, 6, 7503. 25790133 10.1038/ncomms7503 PMC4382999

[93] C. Zhang , D. Zhao , D. Gu , H. Kim , T. Ling , Y. K. R. Wu , L. J. Guo , Adv. Mater. 2014, 26, 5696. 24943876 10.1002/adma.201306091

[94] C. Zhang , Q. Huang , Q. Cui , C. Ji , Z. Zhang , X. Chen , T. George , S. Zhao , L. J. Guo , ACS Appl. Mater. Interfaces 2019, 11, 27216. 31282144 10.1021/acsami.9b08289

[95] G. Zhao , S. M. Kim , S. G. Lee , T. S. Bae , C. W. Mun , S. Lee , H. Yu , G. H. Lee , H. S. Lee , M. Song , J. Yun , Adv. Funct. Mater. 2016, 26, 4180.

[96] J. Yun , W. Wang , T. S. Bae , Y. H. Park , Y. C. Kang , D. H. Kim , S. Lee , G. H. Lee , M. Song , J. W. Kang , ACS Appl. Mater. Interfaces 2013, 5, 9933. 24060352 10.1021/am401845n

[97] D. Zhao , C. Zhang , H. Kim , L. J. Guo , Adv. Energy Mater. 2015, 5, 768.

[98] J.‐Y. Park , H.‐K. Kim , RSC Adv. 2018, 8, 36549. 35558946 10.1039/c8ra07033d PMC9088752

[99] H. Wang , C. Ji , C. Zhang , Y. Zhang , Z. Zhang , Z. Lu , J. Tan , L. J. Guo , ACS Appl. Mater. Interfaces 2019, 11, 11782. 30817123 10.1021/acsami.9b00716

[100] J. Liu , D. Jia , J. M. Gardner , E. M. J. Johansson , X. Zhang , Mater. Today Energy 2019, 13, 152.

[101] Y. Choi , C. S. Kim , S. Jo , Materials 2018, 11, 2231.30423950

[102] A. A. Yousefi , SN Appl. Sci. 2019, 1, 440.

[103] G. Khanarian , J. Joo , X.‐Q. Liu , P. Eastman , D. Werner , K. O'Connell , P. Trefonas , J. Appl. Phys. 2013, 114, 024302.

[104] D. Kojda , R. Mitdank , M. Handwerg , A. Mogilatenko , M. Albrecht , Z. Wang , J. Ruhhammer , M. Kroener , P. Woias , S. F. Fischer , Phys. Rev. B – Condensed Matter Mater. Phys. 2015, 91, 024302.

[105] J. Liang , L. Li , K. Tong , Z. Ren , W. Hu , X. Niu , Y. Chen , Q. Pei , ACS Nano 2014, 8, 1590. 24471886 10.1021/nn405887k

[106] Y. Liu , Q. Chang , L. Huang , J. Mater. Chem. C 2013, 1, 2970.

[107] I. K. Moon , J. I. Kim , H. Lee , K. Hur , W. C. Kim , H. Lee , Sci. Rep. 2013, 3, 1112.

[108] W. Gaynor , G. F. Burkhard , M. D. McGehee , P. Peumans , Adv. Mater. 2011, 23, 2905. 21538594 10.1002/adma.201100566

[109] P. Lee , J. Lee , H. Lee , J. Yeo , S. Hong , K. H. Nam , D. Lee , S. S. Lee , S. H. Ko , Adv. Mater. 2012, 24, 3326. 22610599 10.1002/adma.201200359

[110] J.‐Y. Lee , S. T. Connor , Y. Cui , P. Peumans , Nano Lett. 2008, 8, 689. 18189445 10.1021/nl073296g

[111] J. Krantz , M. Richter , S. Spallek , E. Spiecker , C. J. Brabec , Adv. Funct. Mater. 2011, 21, 4784.

[112] X.‐Y. Zeng , Q.‐K. Zhang , R.‐M. Yu , C.‐Z. Lu , Adv. Mater. 2010, 22, 4484. 20683862 10.1002/adma.201001811

[113] T. C. Hauger , S. M. I. Al‐Rafia , J. M. Buriak , ACS Appl. Mater. Interfaces 2013, 5, 12663. 24224863 10.1021/am403986f

[114] D. Lordan , M. Burke , M. Manning , A. Martin , A. Amann , D. O'Connell , R. Murphy , C. Lyons , A. J. Quinn , ACS Appl. Mater. Interfaces 2017, 9, 4932. 28080027 10.1021/acsami.6b12995

[115] Y. U. Cho , J. Y. Lee , U.‐J. Jeong , S. H. Park , S. L. Lim , K. Y. Kim , J. W. Jang , J. H. Park , H. W. Kim , H. Shin , H. Jeon , Y. M. Jung , I.‐J. Cho , K. J. Yu , Adv. Funct. Mater. 2022, 32, 2105568.

[116] P. D. Donaldson , Z. S. Navabi , R. E. Carter , S. M. L. Fausner , L. Ghanbari , T. J. Ebner , S. L. Swisher , S. B. Kodandaramaiah , Adv. Healthc. Mater. 2022, 11, 2200626. 35869830 10.1002/adhm.202200626 PMC9573805

[117] M. A. Shinde , D.‐J. Lee , B.‐J. Kim , H. Kim , Thin Solid Films 2019, 685, 366.

[118] E. Auroux , G. Huseynova , J. Ràfols‐Ribé , V. M. L. Hera , L. Edman , RSC Adv. 2023, 13, 16943. 37288374 10.1039/d3ra02520a PMC10242295

[119] R. Guo , Y. Yu , Z. Xie , X. Liu , X. Zhou , Y. Gao , Z. Liu , F. Zhou , Y. Yang , Z. Zheng , Adv. Mater. 2013, 25, 3343. 23670964 10.1002/adma.201301184

[120] Y. Yu , X. Xiao , Y. Zhang , K. Li , C. Yan , X. Wei , L. Chen , H. Zhen , H. Zhou , S. Zhang , Z. Zheng , Adv. Mater. 2016, 28, 4926. 27074139 10.1002/adma.201505119

[121] Y. H. Liu , J. L. Xu , S. Shen , X. L. Cai , L. S. Chen , S. D. Wang , J. Mater. Chem. A 2017, 5, 9032.

[122] X. Karagiorgis , D. Shakthivel , G. Khandelwal , R. Ginesi , P. J. Skabara , R. Dahiya , ACS Appl. Mater. Interfaces 2024, 16, 19551. 38567787 10.1021/acsami.4c00682 PMC11040574

[123] A. De Mori , R. S. Jones , M. Cretella , G. Cerri , R. R. Draheim , E. Barbu , G. Tozzi , M. Roldo , Int. J. Mol. Sci. 2020, 21, 2303. 32225118 10.3390/ijms21072303 PMC7178261

[124] J. V. Dcosta , D. Ochoa , S. Sanaur , Adv. Electron. Mater. 2024, 10, 2300559.

[125] Y. Qin , L. Yao , F. Zhang , R. Li , Y. Chen , Y. Chen , T. Cheng , W. Lai , B. Mi , X. Zhang , W. Huang , ACS Appl. Mater. Interfaces 2022, 14, 38021. 35959592 10.1021/acsami.2c09153

[126] T.‐Y. Qu , L.‐J. Zuo , J.‐D. Chen , X. Shi , T. Zhang , L. Li , K.‐C. Shen , H. Ren , S. Wang , F.‐M. Xie , Y.‐Q. Li , A. K.‐Y. Jen , J.‐X. Tang , Adv. Opt. Mater. 2020, 8, 2000669.

[127] J. Yao , B. Qiu , Z.‐G. Zhang , L. Xue , R. Wang , C. Zhang , S. Chen , Q. Zhou , C. Sun , C. Yang , M. Xiao , L. Meng , Y. Li , Nat. Commun. 2020, 11, 2726. 32483159 10.1038/s41467-020-16509-w PMC7264349

[128] X. Chen , G. Xu , G. Zeng , H. Gu , H. Chen , H. Xu , H. Yao , Y. Li , J. Hou , Y. Li , Adv. Mater. 2020, 32, 1908478. 10.1002/adma.201908478 32103580

[129] Y. Sun , M. Chang , L. Meng , X. Wan , H. Gao , Y. Zhang , K. Zhao , Z. Sun , C. Li , S. Liu , H. Wang , J. Liang , Y. Chen , Nat. Electron. 2019, 2, 513.

[130] M. Kaltenbrunner , G. Adam , E. D. Głowacki , M. Drack , R. Schwödiauer , L. Leonat , D. H. Apaydin , H. Groiss , M. C. Scharber , M. S. White , N. S. Sariciftci , S. Bauer , Nat. Mater. 2015, 14, 1032. 26301766 10.1038/nmat4388

[131] Q. Xu , T. Song , W. Cui , Y. Liu , W. Xu , S.‐T. Lee , B. Sun , ACS Appl. Mater. Interfaces 2015, 7, 3272. 25599588 10.1021/am508006q

[132] T. Zhu , Y. Yang , X. Yao , Z. Huang , L. Liu , W. Hu , X. Gong , ACS Appl. Mater. Interfaces 2020, 12, 15456. 32154700 10.1021/acsami.9b22891

[133] J. Wan , Y. Xia , J. Fang , Z. Zhang , B. Xu , J. Wang , L. Ai , W. Song , K. N. Hui , X. Fan , Y. Li , Nano‐Micro Lett. 2021, 13, 44. 10.1007/s40820-020-00566-3 PMC8187532 34138225

[134] Y. Li , S. Wang , J. Guo , R. Chen , F. Zhao , Y. Liu , Carbohydr. Polym. 2019, 219, 21. 31151518 10.1016/j.carbpol.2019.05.016

[135] D. d Ateh , H. a Navsaria , P. Vadgama , J. R. Soc., Interface 2006, 3, 741. 17015302 10.1098/rsif.2006.0141 PMC1885362

[136] D. Thanasamy , D. Jesuraj , S. K. Konda kannan , V. Avadhanam , Polymer 2019, 175, 32.

[137] G. Brunin , F. Ricci , V.‐A. Ha , G.‐M. Rignanese , G. Hautier , npj Comput. Mater. 2019, 5, 1.

[138] E. C. Garnett , B. Ehrler , A. Polman , E. Alarcon‐Llado , ACS Photonics 2021, 8, 61. 33506072 10.1021/acsphotonics.0c01045 PMC7821300

[139] W. Jaffray , S. Saha , V. M. Shalaev , A. Boltasseva , M. Ferrera , Adv. Opt. Photon. 2022, 14, 148.

[140] A. Kumar , N. Kumar , Mater. Today 2023, 10.1016/j.matpr.2023.07.211.

[141] R. M. Mutiso , K. I. Winey , Prog. Polym. Sci. 2015, 40, 63.

[142] W. K. Tan , Y. Matsubara , A. Yokoi , G. Kawamura , A. Matsuda , I. Sugiyama , N. Shibata , Y. Ikuhara , H. Muto , Adv. Powder Technol. 2022, 33, 103528.

[143] A. Tanvir , P. Sobolčiak , A. Popelka , M. Mrlik , Z. Spitalsky , M. Micusik , J. Prokes , I. Krupa , Polymers (Basel) 2019, 11, 1272. 31370311 10.3390/polym11081272 PMC6723293

[144] T. Wang , K. Lu , Z. Xu , Z. Lin , H. Ning , T. Qiu , Z. Yang , H. Zheng , R. Yao , J. Peng , Crystals 2021, 11, 511.

[145] Y.‐H. Ji , Y. Liu , Y.‐Q. Li , H.‐M. Xiao , S.‐S. Du , J.‐Y. Zhang , N. Hu , S.‐Y. Fu , Compos. Sci. Technol. 2016, 132, 57.

[146] N. K. C. Sekhar Rout , RSC Adv. 2021, 11, 5659. 35686160 10.1039/d0ra07800j PMC9133880

[147] R. Balint , N. J. Cassidy , S. H. Cartmell , Acta Biomater. 2014, 10, 2341. 24556448 10.1016/j.actbio.2014.02.015

[148] M. B. Lee , C. T. Lee , W. W. F. Chong , S. M. Sanip , IIUM Eng. J. 2023, 24, 170.

[149] J. Wang , X. Liang , J. Xie , X. Yin , J. Chen , T. Gu , Y. Mo , J. Zhao , S. Liu , D. Yu , J. Zhang , L. Hou , Nanomaterials 2022, 12, 3987. 36432273 10.3390/nano12223987 PMC9693524

[150] C. Zhang , M. O. G. Nayeem , Z. Wang , X. Pu , C. Dagdeviren , Z. L. Wang , X. Zhang , R. Liu , Prog. Mater. Sci. 2023, 138, 101156.

[151] L. Zhao , Q. Ling , X. Fan , H. Gu , ACS Appl. Mater. Interfaces 2023, 15, 40975. 37584619 10.1021/acsami.3c08052

[152] D. Lee , H. Lee , Y. Ahn , Y. Jeong , D.‐Y. Lee , Y. Lee , Nanoscale 2013, 5, nr02320f. 10.1039/c3nr02320f 23842732

[153] K. Mallikarjuna , H. Kim , ACS Appl. Mater. Interfaces 2019, 11, 1969. 30571910 10.1021/acsami.8b14086

[154] P. H. H. Araújo , C. Sayer , R. Giudici , J. G. R. Poço , Polym. Eng. Sci. 2002, 42, 1442.

[155] T. Das , B. K. Sharma , A. K. Katiyar , J.‐H. Ahn , J. Semicond. 2018, 39, 011007.

[156] H. Shin , B. K. Sharma , S. W. Lee , J.‐B. Lee , M. Choi , L. Hu , C. Park , J. H. Choi , T. W. Kim , J.‐H. Ahn , ACS Appl. Mater. Interfaces 2019, 11, 14222. 30912424 10.1021/acsami.8b22135

[157] S. Afroj , S. Tan , A. M. Abdelkader , K. S. Novoselov , N. Karim , Adv. Funct. Mater. 2020, 30, 2000293.

[158] D. Govindarajan , S. Saravanan , S. Sudhakar , S. Vimalraj , ACS Omega 2023, 9, 67. 38222554 10.1021/acsomega.3c07062 PMC10785094

[159] I. Zare , M. Mirshafiei , B. Kheilnezhad , B. F. Far , M. Hassanpour , E. Pishbin , S. S. Eftekhar Vaghefi , F. Yazdian , H. Rashedi , A. Hasan , X. Wang , M. Adeli , P. Makvandi , Carbon 2024, 223, 118970.

[160] A. Esteghamat , O. Akhavan , Microelectron. Eng. 2023, 267–268, 111899.

[161] N. Gupta , S. M. Gupta , S. K. Sharma , Carbon Lett. 2019, 29, 419.

[162] S. N. Habisreutinger , T. Leijtens , G. E. Eperon , S. D. Stranks , R. J. Nicholas , H. J. Snaith , Nano Lett. 2014, 14, 5561. 25226226 10.1021/nl501982b

[163] K. Aitola , K. Sveinbjörnsson , J.‐P. Correa‐Baena , A. Kaskela , A. Abate , Y. Tian , E. M. J. Johansson , M. Grätzel , E. I. Kauppinen , A. Hagfeldt , G. Boschloo , Energy Environ. Sci. 2016, 9, 461.

[164] R. Paul , M. Vincent , V. Etacheri , A. K. Roy , in Carbon Based Nanomaterials for Advanced Thermal and Electrochemical Energy Storage and Conversion (Eds.: R. Paul , V. Etacheri , Y. Wang , C.‐T. Lin ), Elsevier, Amsterdam, Netherlands 2019, pp. 1–24.

[165] L. Hu , D. Hecht , Appl. Phys. Lett. 2009, 94, 3075067.

[166] X. Fan , Adv. Funct. Mater. 2021, 31, 2102717. 10.1002/adfm.202010556 PMC8376022 34421476

[167] J. Han , S. Yuan , L. Liu , X. Qiu , H. Gong , X. Yang , C. Li , Y. Hao , B. Cao , J. Mater. Chem. A 2015, 3, 5375.

[168] J. Li , L. Hu , L. Wang , Y. Zhou , G. Grüner , T. J. Marks , Nano Lett. 2006, 6, 2472. 17090076 10.1021/nl061616a

[169] D. Simien , J. A. Fagan , W. Luo , J. F. Douglas , K. Migler , J. Obrzut , ACS Nano 2008, 2, 1879. 19206428 10.1021/nn800376x

[170] F. Du , J. E. Fischer , K. I. Winey , Phys. Rev. B 2005, 72, 121404.

[171] I. Balberg , N. Binenbaum , Phys. Rev. B 1983, 28, 3799.

[172] M. S. R. N. Kiran , U. Ramamurty , A. Misra , Nanotechnology 2013, 24, 015707. 23221348 10.1088/0957-4484/24/1/015707

[173] D. Hecht , L. Hu , G. Grüner , Appl. Phys. Lett. 2006, 89, 133112.

[174] A. Behnam , L. Noriega , Y. Choi , Z. Wu , A. G. Rinzler , A. Ural , Appl. Phys. Lett. 2006, 89, 093107.

[175] H.‐Z. Geng , K. Kang , K. Lee , G. Yong , H. Choi , D. Lee , K. An , Y. H. Lee , Y. Chang , B. Kim , Y. Lee , NANO: Brief Rep. Rev. 2007, 2, 157.

[176] M. Kaempgen , G. S. Duesberg , S. Roth , Appl. Surf. Sci. 2005, 252, 425.

[177] S. Imani , A. J. Bandodkar , A. M. V. Mohan , R. Kumar , S. Yu , J. Wang , P. P. Mercier , Nat. Commun. 2016, 7, 11650. 27212140 10.1038/ncomms11650 PMC4879240

[178] A. Kaskela , A. G. Nasibulin , M. Y. Timmermans , B. Aitchison , A. Papadimitratos , Y. Tian , Z. Zhu , H. Jiang , D. P. Brown , A. Zakhidov , E. I. Kauppinen , Nano Lett. 2010, 10, 4349. 20863125 10.1021/nl101680s

[179] C. Journet , M. Picher , V. Jourdain , Nanotechnology 2012, 23, 142001. 22433510 10.1088/0957-4484/23/14/142001

[180] K. Fujisawa , H. J. Kim , S. H. Go , H. Muramatsu , T. Hayashi , M. Endo , T. C. Hirschmann , M. S. Dresselhaus , Y. A. Kim , P. T. Araujo , Appl. Sci. 2016, 6, 109.

[181] D. Kuzum , H. Takano , E. Shim , J. C. Reed , H. Juul , A. G. Richardson , J. de Vries , H. Bink , M. A. Dichter , T. H. Lucas , D. A. Coulter , E. Cubukcu , B. Litt , Nat. Commun. 2014, 5, 5259. 25327632 10.1038/ncomms6259 PMC4331185

[182] Y. S. Woo , Micromachines 2019, 10, 13.

[183] K. Kostarelos , K. S. Novoselov , Nat. Nanotech 2014, 9, 744. 10.1038/nnano.2014.224 25286265

[184] Y.‐W. Son , M. L. Cohen , S. G. Louie , Nature 2006, 444, 347. 17108960 10.1038/nature05180

[185] Y. Zhang , V. W. Brar , C. Girit , A. Zettl , M. F. Crommie , Nat. Phys. 2009, 5, 722.

[186] A. R. Urade , I. Lahiri , K. S. Suresh , JOM 2023, 75, 614. 36267692 10.1007/s11837-022-05505-8 PMC9568937

[187] S. Bae , H. Kim , Y. Lee , X. Xu , J.‐S. Park , Y. Zheng , J. Balakrishnan , T. Lei , H. Ri Kim , Y. I. Song , Y.‐J. Kim , K. S. Kim , B. Özyilmaz , J.‐H. Ahn , B. H. Hong , S. Iijima , Nat. Nanotech 2010, 5, 574. 10.1038/nnano.2010.132 20562870

[188] T. Kobayashi , M. Bando , N. Kimura , K. Shimizu , K. Kadono , N. Umezu , K. Miyahara , S. Hayazaki , S. Nagai , Y. Mizuguchi , Y. Murakami , D. Hobara , Appl. Phys. Lett. 2013, 102, 023112.

[189] R. R. Nair , P. Blake , A. N. Grigorenko , K. S. Novoselov , T. J. Booth , T. Stauber , N. M. R. Peres , A. K. Geim , Science 2008, 320, 1308. 18388259 10.1126/science.1156965

[190] H. Liu , Y. Liu , D. Zhu , J. Mater. Chem. 2011, 21, 3335.

[191] S. Pinilla , J. Coelho , K. Li , J. Liu , V. Nicolosi , Nat. Rev. Mater. 2022, 7, 717.

[192] A. Takemoto , T. Araki , K. Nishimura , M. Akiyama , T. Uemura , K. Kiriyama , J. M. Koot , Y. Kasai , N. Kurihira , S. Osaki , S. Wakida , J. M. J. den Toonder , T. Sekitani , Adv. Sci. 2023, 10, 2204746. 10.1002/advs.202204746 PMC9839865 36373679

[193] Q. Niu , K. Gao , Q. Tang , L. Wang , L. Han , H. Fang , Y. Zhang , S. Wang , L. Wang , Carbon 2017, 123, 290.

[194] W. Wu , M. Liu , Y. Gu , B. Guo , H. Ma , P. Wang , X. Wang , R. Zhang , Chem. Eng. J. 2020, 381, 122592.

[195] H. Tang , P. He , T. Huang , Z. Cao , P. Zhang , G. Wang , X. Wang , G. Ding , X. Xie , Carbon 2019, 143, 559.

[196] D. Parviz , F. Irin , S. A. Shah , S. Das , C. B. Sweeney , M. J. Green , Adv. Mater. 2016, 28, 8796. 27546380 10.1002/adma.201601889

[197] I. A. Kinloch , J. Suhr , J. Lou , R. J. Young , P. M. Ajayan , Science 2018, 362, 547. 30385571 10.1126/science.aat7439

[198] H. Lee , M. Kim , I. Kim , H. Lee , Adv. Mater. 2016, 28, 4541. 26823085 10.1002/adma.201505559

[199] I. N. Kholmanov , C. W. Magnuson , R. Piner , J.‐Y. Kim , A. E. Aliev , C. Tan , T. Y. Kim , A. A. Zakhidov , G. Sberveglieri , R. H. Baughman , R. S. Ruoff , Adv. Mater. 2015, 27, 3053. 25866261 10.1002/adma.201500785

[200] V. C. Tung , L.‐M. Chen , M. J. Allen , J. K. Wassei , K. Nelson , R. B. Kaner , Y. Yang , Nano Lett. 2009, 9, 1949. 19361207 10.1021/nl9001525

[201] A. Kelly , D. O'Suilleabhain , C. Gabbett , J. Coleman , Nat. Rev. Mater. 2021, 7, 1.

[202] J. Miao , H. Liu , Y. Li , X. Zhang , ACS Appl. Mater. Interfaces 2018, 10, 23037. 29905073 10.1021/acsami.8b04291

[203] H. Chang , G. Wang , A. Yang , X. Tao , X. Liu , Y. Shen , Z. Zheng , Adv. Funct. Mater. 2010, 20, 2893

[204] Y. Zhao , T. Heumueller , J. Zhang , J. Luo , O. Kasian , S. Langner , C. Kupfer , B. Liu , Y. Zhong , J. Elia , A. Osvet , J. Wu , C. Liu , Z. Wan , C. Jia , N. Li , J. Hauch , C. Brabec , Nat. Energy 2022, 7, 144.

[205] S. Huang , Y. Liu , Y. Zhao , Z. Ren , C. F. Guo , Adv. Funct. Mater. 2019, 29, 1805924.

[206] L. Wen , F. Li , H.‐M. Cheng , Adv. Mater. 2016, 28, 4306. 26748581 10.1002/adma.201504225

[207] K. S. Kim , Y. Zhao , H. Jang , S. Y. Lee , J. M. Kim , K. S. Kim , J.‐H. Ahn , P. Kim , J.‐Y. Choi , B. H. Hong , Nature 2009, 457, 706. 19145232 10.1038/nature07719

[208] N. Liu , A. Chortos , T. Lei , L. Jin , T. R. Kim , W.‐G. Bae , C. Zhu , S. Wang , R. Pfattner , X. Chen , R. Sinclair , Z. Bao , Sci. Adv. 2017, 3, e1700159. 28913422 10.1126/sciadv.1700159 PMC5590784

[209] J. Han , J.‐Y. Lee , J. Lee , J.‐S. Yeo , Adv. Mater. 2018, 30, 1704626.

[210] J. He , R. Zhou , Y. Zhang , W. Gao , T. Chen , W. Mai , C. Pan , Adv. Funct. Mater. 2022, 32, 2107281.

[211] Y. Huang , J. Zhou , P. Ke , X. Guo , C. K. Yiu , K. Yao , S. Cai , D. Li , Y. Zhou , J. Li , T. H. Wong , Y. Liu , L. Li , Y. Gao , X. Huang , H. Li , J. Li , B. Zhang , Z. Chen , H. Zheng , X. Yang , H. Gao , Z. Zhao , X. Guo , E. Song , H. Wu , Z. Wang , Z. Xie , K. Zhu , X. Yu , Nat. Electron. 2023, 6, 1020.

[212] L. Cai , A. Burton , D. A. Gonzales , K. A. Kasper , A. Azami , R. Peralta , M. Johnson , J. A. Bakall , E. B. Villalobos , E. C. Ross , J. A. Szivek , D. S. Margolis , P. Gutruf , Nat. Commun. 2021, 12, 6707. 34795247 10.1038/s41467-021-27003-2 PMC8602388

[213] B. Wang , A. Thukral , Z. Xie , L. Liu , X. Zhang , W. Huang , X. Yu , C. Yu , T. J. Marks , A. Facchetti , Nat. Commun. 2020, 11, 2405. 32415064 10.1038/s41467-020-16268-8 PMC7229221

[214] K. Lee , X. Ni , J. Y. Lee , H. Arafa , D. J. Pe , S. Xu , R. Avila , M. Irie , J. H. Lee , R. L. Easterlin , D. H. Kim , H. U. Chung , O. O. Olabisi , S. Getaneh , E. Chung , M. Hill , J. Bell , H. Jang , C. Liu , J. B. Park , J. Kim , S. B. Kim , S. Mehta , M. Pharr , A. Tzavelis , J. T. Reeder , I. Huang , Y. Deng , Z. Xie , C. R. Davies , et al., Nat. Biomed. Eng. 2020, 4, 148. 31768002 10.1038/s41551-019-0480-6 PMC7035153

[215] W. S. Kim , S. Hong , M. Gamero , V. Jeevakumar , C. M. Smithhart , T. J. Price , R. D. Palmiter , C. Campos , S. I. Park , Nat. Commun. 2021, 12, 157. 33420038 10.1038/s41467-020-20421-8 PMC7794361

[216] L. Nie , A. C. Nusantara , V. G. Damle , R. Sharmin , E. P. P. Evans , S. R. Hemelaar , K. J. van der Laan , R. Li , F. P. Perona Martinez , T. Vedelaar , M. Chipaux , R. Schirhagl , Sci. Adv. 2021, 7, eabf0573. 34138746 10.1126/sciadv.abf0573 PMC8133708

[217] Y. U. Cho , S. L. Lim , J.‐H. Hong , K. J. Yu , npj Flex Electron 2022, 6, 1.

[218] B. S. Kim , K.‐Y. Shin , J. B. Pyo , J. Lee , J. G. Son , S.‐S. Lee , J. H. Park , ACS Appl. Mater. Interfaces 2016, 8, 2582. 26760896 10.1021/acsami.5b10317

[219] J. Park , B. Park , T. Y. Kim , S. Jung , W. J. Choi , J. Ahn , D. H. Yoon , J. Kim , S. Jeon , D. Lee , U. Yong , J. Jang , W. J. Kim , H. K. Kim , U. Jeong , H. H. Kim , C. Kim , Proc. Natl. Acad. Sci. USA 2021, 118, e1920879118.33836558

[220] H. Jin , Z. Zheng , Z. Cui , Y. Jiang , G. Chen , W. Li , Z. Wang , J. Wang , C. Yang , W. Song , X. Chen , Y. Zheng , Nat. Commun. 2023, 14, 4692. 37542045 10.1038/s41467-023-40181-5 PMC10403590

[221] A. Hariri , E. Zhao , A. S. Jeevarathinam , J. Lemaster , J. Zhang , J. V. Jokerst , Sci. Rep. 2019, 9, 11378. 31388020 10.1038/s41598-019-47599-2 PMC6684596

[222] I.‐C. Sun , C.‐H. Ahn , K. Kim , S. Emelianov , JBO 2019, 24, 121903. 31385483 10.1117/1.JBO.24.12.121903 PMC6680094

[223] S. M. Won , H. Wang , B. H. Kim , K. Lee , H. Jang , K. Kwon , M. Han , K. E. Crawford , H. Li , Y. Lee , X. Yuan , S. B. Kim , Y. S. Oh , W. J. Jang , J. Y. Lee , S. Han , J. Kim , X. Wang , Z. Xie , Y. Zhang , Y. Huang , J. A. Rogers , ACS Nano 2019, 13, 10972. 31124670 10.1021/acsnano.9b02030

[224] R. Geng , A. Mena , W. J. Pappas , D. R. McCamey , Nat. Commun. 2023, 14, 1441. 36922502 10.1038/s41467-023-37090-y PMC10017713

[225] D. R. Glenn , K. Lee , H. Park , R. Weissleder , A. Yacoby , M. D. Lukin , H. Lee , R. L. Walsworth , C. B. Connolly , Nat. Methods 2015, 12, 736. 26098019 10.1038/nmeth.3449 PMC4521973

[226] M. Ramezani , J.‐H. Kim , X. Liu , C. Ren , A. Alothman , C. De‐Eknamkul , M. N. Wilson , E. Cubukcu , V. Gilja , T. Komiyama , D. Kuzum , Nat. Nanotechnol. 2024, 19, 504. 38212523 10.1038/s41565-023-01576-z PMC11742260

[227] E. Boto , N. Holmes , J. Leggett , G. Roberts , V. Shah , S. S. Meyer , L. D. Muñoz , K. J. Mullinger , T. M. Tierney , S. Bestmann , G. R. Barnes , R. Bowtell , M. J. Brookes , Nature 2018, 555, 657. 29562238 10.1038/nature26147 PMC6063354

[228] D. Brala , T. Thevathasan , S. Grahl , S. Barrow , M. Violano , H. Bergs , A. Golpour , P. Suwalski , W. Poller , C. Skurk , U. Landmesser , B. Heidecker , J. Am. Heart Assoc. 2023, 12, e027619. 36744683 10.1161/JAHA.122.027619 PMC10111485

[229] O. Feys , P. Corvilain , A. Aeby , C. Sculier , N. Holmes , M. Brookes , S. Goldman , V. Wens , X. De Tiège , Radiology 2022, 304, 429. 35503013 10.1148/radiol.212453

[230] I. Fescenko , A. Laraoui , J. Smits , N. Mosavian , P. Kehayias , J. Seto , L. Bougas , A. Jarmola , V. M. Acosta , Phys. Rev. Appl. 2019, 11, 034029. 31245433 10.1103/PhysRevApplied.11.034029 PMC6594715

[231] S. Cho , H. Han , H. Park , S.‐U. Lee , J.‐H. Kim , S. W. Jeon , M. Wang , R. Avila , Z. Xi , K. Ko , M. Park , J. Lee , M. Choi , J.‐S. Lee , W. G. Min , B.‐J. Lee , S. Lee , J. Choi , J. Gu , J. Park , M. S. Kim , J. Ahn , O. Gul , C. Han , G. Lee , S. Kim , K. Kim , J. Kim , C.‐M. Kang , J. Koo , et al., npj Flex Electron 2023, 7, 1.

[232] S.‐H. Lu , M. Samandari , C. Li , H. Li , D. Song , Y. Zhang , A. Tamayol , X. Wang , Sens. Actuators Rep. 2022, 4, 100075.

[233] I. N. Kurochkin , A. V. Eremenko , G. F. Makhaeva , L. V. Sigolaeva , G. G. Dubacheva , R. J. Richardson , in Counteraction to Chemical and Biological Terrorism in East European Countries, (Eds.: C. Dishovsky , A. Pivovarov ), Springer, Netherlands, Dordrecht 2009, pp. 219–229.

[234] V. N. Dhamu , D. C. Poudyal , C. M. Telang , A. Paul , S. Muthukumar , S. Prasad , Electrochem. Sci. Adv. 2022, 2, e2100128.

[235] E. J. Strand , M. J. Palizzi , C. A. Crichton , M. N. Renny , E. Bihar , R. R. McLeod , G. L. Whiting , Sens. Actuators, B 2023, 387, 133763.

[236] M. A. Zahed , D. K. Kim , S. H. Jeong , M. Selim Reza , M. Sharifuzzaman , G. B. Pradhan , H. Song , M. Asaduzzaman , J. Y. Park , ACS Sens. 2023, 8, 2960. 37498214 10.1021/acssensors.3c00148

[237] Y. Song , R. Y. Tay , J. Li , C. Xu , J. Min , E. Shirzaei Sani , G. Kim , W. Heng , I. Kim , W. Gao , Sci. Adv. 2023, 9, eadi6492. 37703361 10.1126/sciadv.adi6492 PMC10499321

[238] K.‐Y. Chun , S. Seo , C.‐S. Han , Adv. Mater. 2022, 34, 2110082. 10.1002/adma.202110082 35178764

[239] E. Shirzaei Sani , C. Xu , C. Wang , Y. Song , J. Min , J. Tu , S. A. Solomon , J. Li , J. L. Banks , D. G. Armstrong , W. Gao , Sci. Adv. 2023, 9, eadf7388. 36961905 10.1126/sciadv.adf7388 PMC10038347

[240] J. R. S. Brownson , in Solar Energy Conversion Systems (Ed.: J. R. S. Brownson ), Academic Press, Boston 2014, pp. 67–97.

[241] Y. Xu , J. Liu , Small 2016, 12, 1400. 26854030 10.1002/smll.201502988

[242] Z. Chen , Z. Wang , J. Wang , S. Chen , B. Zhang , Y. Li , L. Yuan , Y. Duan , Nanomaterials 2023, 13, 25.

[243] H. Kim , E. Kim , C. Choi , W.‐H. Yeo , Micromachines 2022, 13, mi13040629. 10.3390/mi13040629 PMC9029742 35457934

[244] Z. Chen , Z. Lin , S. N. Obaid , E. Rytkin , S. A. George , C. Bach , M. Madrid , M. Liu , J. LaPiano , A. Fehr , X. Shi , N. Quirion , B. Russo , H. Knight , A. Aduwari , I. R. Efimov , L. Lu , Sci. Adv. 2023, 9, eadi0757. 37406128 10.1126/sciadv.adi0757 PMC10321742

[245] J. Kim , M. Patel , H.‐S. Kim , SPIE Newsroom 2016, 1, 006406.

[246] A. Mascia , A. Spanu , A. Bonfiglio , P. Cosseddu , Sci. Rep. 2023, 13, 16232. 37758843 10.1038/s41598-023-43360-y PMC10533849

[247] S. Manohar , D. Razansky , Adv. Opt. Photon. 2016, 8, 586.

[248] H. Chen , S. Agrawal , M. Osman , J. Minotto , S. Mirg , J. Liu , A. Dangi , Q. Tran , T. Jackson , S.‐R. Kothapalli , BME Frontiers 2022, 2022, 9871098. 37850172 10.34133/2022/9871098 PMC10521654

[249] X. Gao , X. Chen , H. Hu , X. Wang , W. Yue , J. Mu , Z. Lou , R. Zhang , K. Shi , X. Chen , M. Lin , B. Qi , S. Zhou , C. Lu , Y. Gu , X. Yang , H. Ding , Y. Zhu , H. Huang , Y. Ma , M. Li , A. Mishra , J. Wang , S. Xu , Nat. Commun. 2022, 13, 7757. 36522334 10.1038/s41467-022-35455-3 PMC9755152

[250] E. Z. Zhang , B. Povazay , J. Laufer , A. Alex , B. Hofer , B. Pedley , C. Glittenberg , B. Treeby , B. Cox , P. Beard , W. Drexler , Biomed. Opt. Express 2011, 2, 2202. 21833358 10.1364/BOE.2.002202 PMC3149519

[251] S. Cho , M. Kim , J. Ahn , Y. Kim , J. Lim , J. Park , H. H. Kim , W. J. Kim , C. Kim , Nat. Commun. 2024, 15, 1444. 38365897 10.1038/s41467-024-45273-4 PMC10873420

[252] H. Chen , M. Osman , S. Mirg , S. Agrawal , J. Cai , A. Dangi , S.‐R. Kothapalli , in Photons Plus Ultrasound: Imaging and Sensing 2021, SPIE, XX 2021, pp. 142–149.

[253] D. Kim , E. Park , J. Park , B. Perleberg , S. Jeon , J. Ahn , M. Ha , H. H. Kim , J. Y. Kim , C. K. Jung , C. Kim , Laser Photonics Rev. 2024, 18, 2300652.

[254] W. Yu , W. C. Jiang , Q. Lin , T. Lu , Nat. Commun. 2016, 7, 12311. 27460277 10.1038/ncomms12311 PMC4974467

[255] M. Xie , X. Yu , L. V. H. Rodgers , D. Xu , I. Chi‐Durán , A. Toros , N. Quack , N. P. de Leon , P. C. Maurer , Proc. Natl. Acad. Sci. USA 2022, 119, e2114186119. 35193961 10.1073/pnas.2114186119 PMC8872777

[256] H. Zhang , H. Chen , J.‐H. Lee , E. Kim , K.‐Y. Chan , H. Venkatesan , X. Shen , J. Yang , J.‐K. Kim , ACS Nano 2023, 17, 5921. 36920071 10.1021/acsnano.3c00015

[257] Y. Lu , Y. Yue , Q. Ding , C. Mei , X. Xu , S. Jiang , S. He , Q. Wu , H. Xiao , J. Han , InfoMat 2023, 5, e12409.

[258] B. Bao , Y. Hua , R. Wang , D. Li , Adv. Quantum Technol. 2023, 6, 2200146.

[259] N. Aslam , H. Zhou , E. K. Urbach , M. J. Turner , R. L. Walsworth , M. D. Lukin , H. Park , Nat. Rev. Phys. 2023, 5, 157. 36776813 10.1038/s42254-023-00558-3 PMC9896461

[260] C.‐J. Yu , S. von Kugelgen , D. W. Laorenza , D. E. Freedman , ACS Cent. Sci. 2021, 7, 712. 34079892 10.1021/acscentsci.0c00737 PMC8161477

[261] D. L. Sage , K. Arai , D. R. Glenn , S. J. DeVience , L. M. Pham , L. Rahn‐Lee , M. D. Lukin , A. Yacoby , A. Komeili , R. L. Walsworth , Nature 2013, 496, 486. 23619694 10.1038/nature12072 PMC3641584

[262] H. C. Davis , P. Ramesh , A. Bhatnagar , A. Lee‐Gosselin , J. F. Barry , D. R. Glenn , R. L. Walsworth , M. G. Shapiro , Nat. Commun. 2018, 9, 131. 29317627 10.1038/s41467-017-02471-7 PMC5760582

[263] P. Wang , S. Chen , M. Guo , S. Peng , M. Wang , M. Chen , W. Ma , R. Zhang , J. Su , X. Rong , F. Shi , T. Xu , J. Du , Sci. Adv. 2019, 5, eaau8038. 30989109 10.1126/sciadv.aau8038 PMC6457937

[264] T. M. Babinec , B. J. M. Hausmann , M. Khan , Y. Zhang , J. R. Maze , P. R. Hemmer , M. Lončar , Nat. Nanotech 2010, 5, 195. 10.1038/nnano.2010.6 20154687

[265] J. F. Barry , J. M. Schloss , E. Bauch , M. J. Turner , C. A. Hart , L. M. Pham , R. L. Walsworth , Rev. Mod. Phys. 2020, 92, 015004.

[266] S. Sengottuvel , M. Mrózek , M. Sawczak , M. J. Głowacki , M. Ficek , W. Gawlik , A. M. Wojciechowski , Sci. Rep. 2022, 12, 17997. 36289436 10.1038/s41598-022-22610-5 PMC9606006

[267] N. Mathes , M. Comas , R. Bleul , K. Everaert , T. Hermle , F. Wiekhorst , P. Knittel , R. A. Sperling , X. Vidal , Nanoscale Adv. 2023, 6, 247. 38125606 10.1039/d3na00684k PMC10729879

[268] B. Heidecker , Nature 2023, 619, 465. 10.1038/d41586-023-02247-8 37464081

[269] L. Shi , Z. Li , M. Chen , Y. Qin , Y. Jiang , L. Wu , Nat. Commun. 2020, 11, 3529. 32669556 10.1038/s41467-020-17298-y PMC7363923

[270] Y. Lu , X. Liu , R. Hattori , C. Ren , X. Zhang , T. Komiyama , D. Kuzum , Adv. Funct. Mater. 2018, 28, 1800002. 34084100 10.1002/adfm.201800002 PMC8172040

[271] M. N. Cecyn , K. P. Abrahao , IBRO Neurosci. Rep. 2023, 15, 143. 38204571 10.1016/j.ibneur.2023.07.003 PMC10776314

[272] “High‐density Electroencephalographic Acquisition in a Rodent Model Using Low‐cost and Open‐source Resources,” can be found under https://app.jove.com/t/54908. 10.3791/54908 PMC5226321 27929470

[273] J. W. Barter , S. Li , T. Sukharnikova , M. A. Rossi , R. A. Bartholomew , H. H. Yin , J. Neurosci. 2015, 35, 2703. 25673860 10.1523/JNEUROSCI.3245-14.2015 PMC4323537

[274] E. K. Unger , J. P. Keller , M. Altermatt , R. Liang , A. Matsui , C. Dong , O. J. Hon , Z. Yao , J. Sun , S. Banala , M. E. Flanigan , D. A. Jaffe , S. Hartanto , J. Carlen , G. O. Mizuno , P. M. Borden , A. V. Shivange , L. P. Cameron , S. Sinning , S. M. Underhill , D. E. Olson , S. G. Amara , D. T. Lang , G. Rudnick , J. S. Marvin , L. D. Lavis , H. A. Lester , V. A. Alvarez , A. J. Fisher , J. A. Prescher , et al., Cell 2020, 183, 1986. 33333022 10.1016/j.cell.2020.11.040 PMC8025677

[275] Z. Liu , X. Lu , V. Villette , Y. Gou , K. L. Colbert , S. Lai , S. Guan , M. A. Land , J. Lee , T. Assefa , D. R. Zollinger , M. M. Korympidou , A. L. Vlasits , M. M. Pang , S. Su , C. Cai , E. Froudarakis , N. Zhou , S. S. Patel , C. L. Smith , A. Ayon , P. Bizouard , J. Bradley , K. Franke , T. R. Clandinin , A. Giovannucci , A. S. Tolias , J. Reimer , S. Dieudonné , F. St‐Pierre , Cell 2022, 185, 3408. 35985322 10.1016/j.cell.2022.07.013 PMC9563101

[276] D. Giasafaki , C. Mitzithra , V. Belessi , T. Filippakopoulou , A. Koutsioukis , V. Georgakilas , G. Charalambopoulou , T. Steriotis , Nanomaterials 2022, 12, 3443. 36234570 10.3390/nano12193443 PMC9565487

[277] A. Vyatskikh , A. Tsapenko , E. Gilshtein , T. Koltsova , T. Larionova , A. Talyzin , A. Anisimov , I. Anoshkin , E. Kauppinen , O. Tolochko , A. Nasibulin , Carbon 2016, 100, 035.

[278] H. M. Saleh , A. I. Hassan , Sustainability 2023, 15, 10891.

[279] M. Nie , B. Li , Y.‐L. Hsieh , K. K. Fu , J. Zhou , ACS Nano 2022, 16, 19810. 36475644 10.1021/acsnano.2c08166

[280] A. Sahasrabudhe , L. E. Rupprecht , S. Orguc , T. Khudiyev , T. Tanaka , J. Sands , W. Zhu , A. Tabet , M. Manthey , H. Allen , G. Loke , M.‐J. Antonini , D. Rosenfeld , J. Park , I. C. Garwood , W. Yan , F. Niroui , Y. Fink , A. Chandrakasan , D. V. Bohórquez , P. Anikeeva , Nat. Biotechnol. 2024, 42, 892. 37349522 10.1038/s41587-023-01833-5 PMC11180606

[281] Z. Dong , W. Li , H. Wang , X. Jiang , H. Liu , L. Zhu , H. Chen , Matter 2022, 5, 448.

[282] S. Choi , S. I. Han , D. Jung , H. J. Hwang , C. Lim , S. Bae , O. K. Park , C. M. Tschabrunn , M. Lee , S. Y. Bae , J. W. Yu , J. H. Ryu , S.‐W. Lee , K. Park , P. M. Kang , W. B. Lee , R. Nezafat , T. Hyeon , D.‐H. Kim , Nat. Nanotech. 2018, 13, 1048. 10.1038/s41565-018-0226-8 30104619

[283] J. Miao , S. Chen , H. Liu , X. Zhang , Chem. Eng. J. 2018, 345, 260.

[284] S. Cataldo , P. Salice , E. Menna , B. Pignataro , Energy Environ. Sci. 2012, 5, 5919.

[285] L. Yu , C. Shearer , J. Shapter , Chem. Rev. 2016, 116, 13413. 27704787 10.1021/acs.chemrev.6b00179

