# Supplementary material for: Recent Advances in Transparent Electrodes and Their Multimodal Sensing Applications
Source: Adv Sci (Weinh). 2024 Aug 9;11(38):2405099. doi: 10.1002/advs.202405099 (PMC11481197; doi:10.1002/advs.202405099)
Supplement: Supplementary file 1 — Supporting Information [file ADVS-11-2405099-s001.pdf]

ADVANCED SCIENCE
Open Access

Supporting Information

for *Adv. Sci.*, DOI 10.1002/advs.202405099

Recent Advances in Transparent Electrodes and Their Multimodal Sensing Applications

*Majed Althumayri, Ritu Das, Ramu Banavath, Levent Beker, Alin M. Achim and Hatice Ceylan Koydemir**

Supporting Information

Recent Advances in Transparent Electrodes and Their Multimodal Sensing Applications

*Majed Althumayri, Ritu Das, Ramu Banavath, Levent Beker, Alin M. Achim and Hatice Ceylan Koydemir**

LIST OF ABBREVIATIONS

1. **Ag**: Silver
2. **AgNWs**: Silver Nanowires
3. **Al**: Aluminum
4. **APTMS**: [3-Aminopropyl]-Trimethoxysilane
5. **AZO**: Aluminum Zinc Oxide
6. **Au**: Gold
7. **BMI**: Brain-Machine Interface
8. **BSA**: Bovine Serum Albumin
9. **Ca2+**: Calcium Ions
10. **CNs**: Carbon Nanomaterials
11. **CNTs**: Carbon Nanotubes
12. **Cs_2CO_3**: Caesium Carbonate
13. **Cu**: Copper
14. **EDMR**: Electrically Detected Magnetic Resonance
15. **ECoG**: Electrocorticography
16. **EMI**: Electromagnetic Interference
17. **F-N**: Fowler-Nordheim
18. **FTEs**: Flexible Transparent Electrodes
19. **FTO**: Fluorine-Doped Tin Oxide

20. **Ga_2O_3**: Gallium(III) Oxide
21. **Ge**: Germanium
22. **GZO**: Gallium Zinc Oxide
23. **HNO_3**: Nitric Acid
24. **IJV**: Internal Jugular Vein
25. **IZO**: Indium-Zinc Oxide
26. **ITO**: Indium Tin Oxide
27. **LEDs**: Light-Emitting Diodes
28. **LiNbO3**: Lithium Niobate
29. **MEAs**: Microelectrode Arrays
30. **MNPs**: Magnetic Nanoparticles
31. **MWCNTs**: Multi-Walled Carbon Nanotubes
32. **MPTMS**: [3-Mercaptopropyl]-Trimethoxysilane
33. **MNWs**: Metal Nanowires
34. **MoO_3**: Molybdenum Trioxide
35. **MUA**: 11-Mercaptoundecanoic Acid
36. **MXene**: Titanium Carbide Ti3C2Tx
37. **Ni**: Nickel
38. **NiO**: Nickel Oxide
39. **Nb_2O_5**: Niobium Pentoxide
40. **NUV**: Near-Ultraviolet
41. **NV**: Nitrogen-Vacancy
42. **OECTs**: Organic Electrochemical Transistors
43. **OLEDs**: Organic Light-Emitting Diodes
44. **ODMR**: Optically Detected Magnetic Resonance
45. **OSC**: Organic Solar Cell

46. **PANI**: Polyaniline

47. **PAM**: Polyacrylamide

48. **PAT**: Photoacoustic Tomography

49. **PDMS**: Polydimethylsiloxane

50. **PEDOT**: Poly(3,4-ethylenedioxythiophene) Polystyrene Sulfonate

51. **PEG**: Polyethylene Glycol

52. **PET**: Polyethylene Terephthalate

53. **PLGA**: Poly(lactic-co-glycolic acid)

54. **PMMA**: Polymethyl Methacrylate

55. **PMN-PT**: Lead Magnesium Niobate-Lead Titanate

56. **PPV**: Poly(phenylene-vinylene)

57. **PPy**: Polypyrrole

58. **PSS**: Polystyrene Sulfonate

59. **PTFE**: Polytetrafluoroethylene

60. **PTh**: Polythiophene

61. **PVDF**: Polyvinylidene Fluoride

62. **PVD**: Physical Vapor Deposition

63. **PVP**: Polyvinyl Pyrrolidone

64. **PZT**: Lead Zirconate Titanate

65. **SEM**: Scanning Electron Microscopy

66. **SERF**: Spin-Exchange Relaxation Free

67. **SnO_2**: Tin(IV) Oxide

68. **SQUID**: Superconducting Quantum Interference Device

69. **SWCNTs**: Single-Walled Carbon Nanotubes

70. **Ta_2O_5**: Tantalum Pentoxide

71. **TCFs**: Transparent Conductive Films

72. **TCEs**: Transparent Carbon Electrodes

73. **TeO_2**: Tellurium Dioxide

74. **TEs**: Transparent Electrodes

75. **TEM**: Transmission Electron Microscope

76. **TFSI**: Trifluoromethylsulfonyl Imide

77. **TiO_2**: Titanium Dioxide

78. **TUT**: Transparent Ultrasound Transducer

79. **UHCS**: Urchin-Like Hollow Carbon Spheres

80. **US**: Ultrasound

81. **USPA**: Ultrasound and Photoacoustic

82. **UV**: Ultraviolet

83. **VCSEL**: Vertical-Cavity Surface-Emitting Laser

84. **XPS**: X-ray Photoelectron Spectroscopy

85. **ZnO**: Zinc Oxide

Table S1: Emerging Metal Oxide-Based Transparent Electrode Materials.

Material & Composition	General Properties & Application Areas	Electrode Structure	Optical Properties	Electrical Properties	Mechanical Properties	Biomedical Application	Substrate	Reference
ITO	Transparency over visible spectrum, low autofluorescence for LEDs, displays, solar cells, microelectrodes	ITO electrode, Au interconnect, Parylene C substrate	90% transparency	30 kΩ	Flexible	BMI, neural circuitry study	Parylene C	[1]
ITO, Au	High-density electrode array for electrical neural recording and optical neural stimulation	ITO electrode, Au traces, and pads	94% transparency	1-5 kΩ	Flexible	Implantable neural device	Polymer	[2]
ITO	Transparent, low-autofluorescent for neuroscience, imaging of Ca 2+ signals, EEG recording	MicroECoG device	93% transparency	345 kΩ	Flexible	Two-photon imaging, EEG recording	Parylene HT	[3]
ZnO, ITO, Pt-Ir wire	High conductivity, optical transparency for optogenetics, probe implantation	Square pillar arrays with ITO	90% transparency	300 kΩ	-	Intracortical microprobe array	ZnO	[4]
AgNWs, IZO	Transparent, flexible, conductive for ECoG electrodes, neural recordings	Hybrid films of AgNWs with IZO	60% transparency	20 kΩ	Flexible, stable under conditions	Brain-machine research	Glass	[5]
SnO_2/ITO	Semitransparent light-emitting electrochemical cells, high transparency for light-emitting devices	Transparent top SnO_2/ITO contact	75% transparency	-	-	-	-	[6]
TiO_2	1D TiO_2 nanorod arrays for electrochromic energy storage, smart devices	TiO_2/PEDOT nanorod film electrode	~55.5%	Shorter response time (~0.5 s)	-	-	FTO glass with TiO_2 nanorods	[7]
ZnO	High conductivity for organic optoelectronic devices	Sol-gel-grown ZnO films	~80%	460 S cm^{-1}	-	-	Glass	[8]
IZO	Resistivity, electron mobility, transparency in visible and NIR ranges for optoelectronic devices, tandem solar cells	-	≥ 77%	Minimum resistance of 3.4×10^{-4} Ω•cm at 100°C	-	-	Soda-lime glass	[9]

ITO, InGaN/AlGaIn N	Transparent electrode for NUV LEDs in optoelectronics, LED technology	Thin film	89.00%	Sheet resistance of 131 Ω/, contact resistance of 3.1 × 10^{-3} Ω•cm^2	-	-	Glass substrate	[10]
Al:ZnO, Ag-NW	Flexible, semitransparent, conductive, high-temperature stability for textile-integrated electronics, sensing applications	Combination of Al:ZnO and Ag-NW layers	20-25%	Sheet resistance of 10 Ω/sq	Withstands mechanical and thermal stress	-	Polyamide-based textile	[11]
SnO_2:F	Transparent conductive oxide electrodes for opto-electrical devices, gas sensors, displays, solar cells	Thin films	Influenced by surface morphology	Influenced by doping	-	-	Glass substrate	[12]
Mixed Molybdenum-Titanium-Oxide (MTO)	MTO layers for solar cells, OLEDs, sensing applications with high water stability and electronic properties	DMD transparent electrodes	-	Low sheet resistance around 5 Ω/sq	-	-	Glass, PET	[13]
TiO_2, TiO_2:Nb, NiO, Ag	High transparency and conductivity for solar cells	Oxide/metal/oxide layers via DC sputtering	-	Low electrical resistivity (~10^{-3} Ω•cm)	-	-	Glass, plastic	[14]

Table S2: Recent Metal-Based Transparent Electrode Materials.

Material & Composition	General Properties & Application Areas	Electrode Structure	Optical Properties	Electrical Properties	Mechanical Properties	Biomedical Application	Substrate	Reference
Nanomesh (Au) and PEDOT:PSS	Transparency for in vivo and two-photon imaging	Nanomesh (Au) and PEDOT:PSS	73% transparency	130 kΩ	Flexible	Imaging of neurons	-	[15]
Au Nanomesh and PEDOT:PSS	High-density electrode array for electrophysiological studies	Penetrating arrays on Parylene C	67% transparency	149 kΩ	Flexible	Neuronal activities monitoring	-	[16]
Au Nanowires on PDMS	High transparency for photonic applications	Au nanowires on PDMS	90% transparency	1 kΩ	Flexible	Two-photon calcium imaging	-	[17]
Au Nanonetwork	High transparency for neural activity recording	Au nanonetwork	81% transparency	34 kΩ	Flexible	In vivo neural activity recording	-	[18]
Au Grid, PEDOT:PSS	Transparency for EEG spatial mapping	Au grid wiring, PEDOT:PSS, SU-8 passivation	69% transparency	10 kΩ	Flexible	EEG recordings mapping	-	[19]
Ag NWs and PLLA	Biodegradable, flexible conductor for heaters, EMI shielding	AgNW micromesh in PLLA	52.8% transparency	~10 Ω/sq	Flexible	-	PTFE	[20]
Gold Coated Ag NWs	Stretchable, transparent for biomedical engineering	MEAs with Au-coated Ag NWs	80.1% transparency	237 kΩ	Stretchable	Dynamic biomedical applications	PDMS	[21]
Nanostructured Au on CNTs in PDMS	Stretchable, transparent electrochemical sensor for H_2O_2 analysis	Nano-Au/CNT networks in PDMS	-	Excellent stability	Flexible	Biomolecule sensing	PDMS	[22]

AgNWs and cPI Varnish	Conductive, transparent electrode for E-skin devices, heaters, sensors	Kirigami patterned AgNWs/cPI composite	>80% transparency	-	Ultrastretchable	Health monitoring	Glass	[23]
Transparent Dielectric Film, Cu Nanoparticles	High conductivity, transparency for optoelectronics	Metallic wires in dielectric film	≈ 60% transparency	-	-	-	Glass	[24]
ITO and Au	Transparent, conductive electrodes for biofilm diagnostics	ITO/Au for microbial electrochemical technologies	-	-	-	Biofilm diagnostics	ITO	[25]
Metal Nanomesh Films: $ZnO/Au/Al_2O_3$	Conductive, transparent, thermochromic for smart windows	Metal nanomesh films	Over 90% transparency	<20 Ω/sq	Flexible	-	TO/elastomeric	[26]
Ultrathin Silver Films on NOA63	Ultrasmooth films for bioelectronics	Template-patterned TEs on NOA63	59% transparency	16 Ω/sq	Resilient to bending	Bioelectronics	Glass with OTS	[27]

Table S3: Recent Conductive Polymer-Based Transparent Electrode Materials.

Material & Composition	General Properties & Application Areas	Electrode Structure	Optical Properties	Electrical Properties	Mechanical Properties	Biomedical Application	Substrate	Reference
PEDOT:PSS	Transparent, highly conductive for neuroscience and biomedical engineering	Array	85% transparency	50 to 56 kΩ	Flexible	Neural implants for electrophysiology and optogenetics	PET	[28]
PEDOT:PSS	Transparent, inkjet-printed electrode arrays for neural sensing and opto-electrophysiological recordings	Ten electrodes over ≈45 mm^2 window	80% and 77% transparency	-	Flexible and conformal	Neural sensing and recordings	PET film	[29]
Emissive Semiconducting Polymer, Ionic Liquid, PEDOT:PSS	Metal-free, transparent in off-state, bright luminance in on-state for light-emitting electrochemical cells	Sandwiched between two PEDOT:PSS electrodes	0.7% transparency	Conductivity of 100 S cm^{-1}	-	-	Glass	[30]
Silver Nanowire	High conductivity and flexibility for touch sensors and electronics	Single-layer, laser-patterned	>80% transmittance	Superior conductivity	Bendable, durable	-	Plastic film	[31]
Silver Nanowire, PEDOT:PSS, ITO	High conductivity, flexibility, thermal stability for film heaters and flexible electronics	Ternary composite layer	95% at 550 nm	Low electrical resistance	Flexible, durable	-	Flexible substrate	[32]
SWCNTs, PEDOT:PSS, iTMC	Solution-processed, flexible for lighting and displays	SWCNTs as anode, PEDOT:PSS layer	High transparency	Effective hole injection	Flexible	-	PET	[33]
Graphene-PEDOT Hybrid	High conductivity, flexibility, processability for optoelectronic devices	Hybrid composite	>80% transmittance in 400-1800 nm	0.2 S/cm	Good flexibility, high thermal stability	-	-	[34]
Superaligned Carbon Nanotubes (SACNT)	High conductivity, flexibility, stretchability for touch panels and displays	Superaligned network of CNTs	Not specified	Low sheet resistance, enhanced by metal deposition	Stretchable, durable	-	Flexible substrates including PET	[35]

Table S4: Recent Graphene and Carbon-Based Transparent Electrode Materials.

Material & Composition	General Properties & Application Areas	Electrode Structure	Optical Properties	Electrical Properties	Mechanical Properties	Biomedical Application	Substrate	Reference
Graphene (4 layers)	Broad spectrum transparency, flexible, tunable properties	Graphene (4 layers)	90% transparency	243 kΩ	Flexible	Evoked potentials, baseline activities recording	-	[36]
Nitric Acid Doped Graphene	Enhanced transparency and conductivity	Nitric acid doped graphene	90% transparency	541 kΩ	-	Electrophysiology and calcium imaging	-	[37]
Graphene with Micropillars	Transparent, glue-free, skin-attachable	Single-layered graphene film on PDMS	70.8% transparency	-	Measures skin elasticity	Skin-elasticity measurement, monitoring	PDMS	[38]
Carbon and FTO	Optimal electrochemical parameters, high stability	-	80-84.5% transparency	-	-	Electrochemical sensors	Silicon or glass	[39]
SWNTs and PEDOT on PDMS	Stretchable, transparent, electrochemical	SWNTs@PEDOT/PDMS film	81.5% transparency	82 Ω	Highly compliant	Biochemical monitoring from stretched cells	PDMS	[40]
Graphene Arrays	Transparent, conformable for cardiac diagnosis	Graphene arrays	~97%	Low interface impedance, high charge capacity	Tissue-conformable	Cardiac arrhythmia diagnosis	PMMA	[41]
Graphene and Platinum Nanoparticles	Low impedance, high transmittance for neuroscience	Transparent graphene microelectrodes with Pt nanoparticles	0.9% transparency	Impedance reduction	-	Electrical recording and optical imaging in neuroscience	PET	[42]
P(VDF-TrFE) Nanofibers and SWCNTs	Transparent, flexible piezocomposite with enhanced properties	SWCNT film as transparent electrode	70% transparency	-	Enhanced due to 1D nanomaterials	Wearable self-powered electronics	-	[43]

Graphene Films	High-quality, wafer-scale graphene	Wafer-scale graphene layers	High transparency	High electron mobility	Flexible, stretchable	-	Transferable to arbitrary substrates, including PET and rubber	[44]
Graphene-PEDOT Hybrid	High conductivity, flexibility, processability	Hybrid composite	>80% transmittance in 400-1800 nm	0.2 S/cm	Good flexibility, high thermal stability	-	-	[45]

Table S5: Cost-Effectiveness of Transparent Electrode Materials

**Material Type**	**Example Materials**	**Cost Factors**	**Economic Benefits**	**References**
Metal Oxide-Based	ITO, ZnO, AZO, FTO	High cost of indium (ITO), energy-intensive production	Lower-cost alternatives like ZnO and AZO, abundant materials	[46–48]
Metal-Based	AgNWs, AuNWs, CuNWs	High cost of silver and gold, cheaper alternatives like copper	Excellent conductivity and flexibility, ongoing research to reduce costs	[49–51]
Conductive Polymer-Based	PEDOT	Lower material costs, simpler processing techniques	Scalable for mass production, challenges in consistency and stability Scalable for mass production, challenges in consistency and stability	[52–54]
Carbon-Based	Graphene, CNTs	Historically high production costs, reducing with advancements	Balance of performance and cost, potential for large-scale production	[55–57]

Table S6: Comparison of Properties of Transparent Electrodes to Conventional Electrodes

Property	Transparent Electrodes	Conventional Electrodes	References
Optical Transparency	High optical transparency (up to 90% or more)	Opaque or low transparency	[58–60]
Electrical Conductivity	Comparable to conventional electrodes	High electrical conductivity	[53,61]
Mechanical Flexibility	High flexibility, can be bent and stretched without losing functionality	Generally rigid and brittle	[46,62,63]
Material Cost	Potentially lower cost	Often higher cost	[48,64]
Environmental Impact	Environmentally friendly, with potential for using abundant materials	Limited environmental sustainability, often relying on rare or toxic elements	[65,66]
Biocompatibility	High biocompatibility, suitable for biomedical applications	Generally lower biocompatibility	[67–69]

References:

[1] P. Ledochowitsch, E. Olivero, T. Blanche, M. M. Maharbiz, *Annu Int Conf IEEE Eng Med Biol Soc* **2011**, *2011*, 2937.
[2] K. Y. Kwon, B. Sirowatka, A. Weber, W. Li, *IEEE Transactions on Biomedical Circuits and Systems* **2013**, *7*, 593.
[3] A. Zatonyi, M. Madarász, Á. Szabó, T. Lőrincz, R. Hodován, B. Rózsa, Z. Fekete, *Journal of Neural Engineering* **2019**, *17*, DOI 10.1088/1741-2552/ab603f.
[4] J. Lee, I. Ozden, Y.-K. Song, A. V. Nurmikko, *Nat Methods* **2015**, *12*, 1157.
[5] J. P. Neto, A. Costa, J. Vaz Pinto, A. Marques-Smith, J. C. Costa, R. Martins, E. Fortunato, A. R. Kampff, P. Barquinha, *ACS Appl. Nano Mater.* **2021**, *4*, 5737.
[6] L. Mardegan, A. Paliwal, K. P. S. Zanoni, D. Tordera, H. J. Bolink, *Advanced Optical Materials* **2022**, *10*, 2201953.
[7] B. Zhuang, Q. Zhang, K. Zhou, H. Wang, *RSC Adv.* **2023**, *13*, 18229.
[8] Z. Chen, J. Wang, H. Wu, J. Yang, Y. Wang, J. Zhang, Q. Bao, M. Wang, Z. Ma, W. Tress, Z. Tang, *Nat Commun* **2022**, *13*, 4387.
[9] A. K. Akhmedov, E. K. Murliev, A. S. Asvarov, A. E. Muslimov, V. M. Kanevsky, *Coatings* **2022**, *12*, 1583.
[10] M.-J. Kim, *Materials* **2023**, *16*, 4718.
[11] M. L. Hupfer, A. Gawlik, J. Dellith, J. Plentz, *Materials* **2023**, *16*, 3961.
[12] K. Juraić, P. Dubček, M. Bohač, A. Gajović, S. Bernstorff, M. Čeh, A. Hodzic, D. Gracin, *Materials* **2022**, *15*, 4814.
[13] S. Goetz, R. A. Wibowo, M. Bauch, N. Bansal, G. Ligorio, E. List-Kratochvil, C. Linke, E. Franzke, J. Winkler, M. Valtiner, T. Dimopoulos, *J Mater Sci* **2022**, *57*, 8752.
[14] L. Hrostea, P. Lisnic, R. Mallet, L. Leontie, M. Girtan, *Nanomaterials (Basel)* **2021**, *11*, 1416.
[15] Y. Qiang, P. Artoni, K. J. Seo, S. Culaclii, V. Hogan, X. Zhao, Y. Zhong, X. Han, P.-M. Wang, Y.-K. Lo, Y. Li, H. A. Patel, Y. Huang, A. Sambangi, J. S. V. Chu, W. Liu, M. Fagiolini, H. Fang, *Science Advances* **2018**, *4*, eaat0626.
[16] K. J. Seo, P. Artoni, Y. Qiang, Y. Zhong, X. Han, Z. Shi, W. Yao, M. Fagiolini, H. Fang, *Advanced Biosystems* **2019**, *3*, 1800276.
[17] A. F. Renz, J. Lee, K. Tybrandt, M. Brzezinski, D. A. Lorenzo, M. Cerra Cheraka, J. Lee, F. Helmchen, J. Vörös, C. M. Lewis, *Adv Healthc Mater* **2020**, *9*, e2000814.

[18] J. Seo, K. Kim, K. Seo, M. Kim, S. Jeong, H. Kim, J. Ghim, J. H. Lee, N. Choi, J.-Y. Lee, H. Lee, *Advanced Functional Materials* **2020**, *30*, 2000896.
[19] W. Lee, D. Kim, N. Matsuhisa, M. Nagase, M. Sekino, G. G. Malliaras, T. Yokota, T. Someya, *Proc Natl Acad Sci U S A* **2017**, *114*, 10554.
[20] W. Hong, B. Sun, Z. Li, Z. Fu, J. Zhang, M. Jiang, Y. Zhang, Y. Li, Y. Zhang, K. Qian, *ACS Appl. Electron. Mater.* **2022**, *4*, 5446.
[21] Z. Chen, K. Nguyen, G. Kowalik, X. Shi, J. Tian, M. Doshi, B. R. Alber, X. Ning, M. W. Kay, L. Lu, *Transparent and Stretchable Metal Nanowire Composite Recording Microelectrode Arrays*, Bioengineering, **2022**.
[22] J. Li, M. Jiang, M. Su, L. Tian, W. Shi, C. Yu, *Anal. Chem.* **2021**, *93*, 6723.
[23] P. Won, J. J. Park, T. Lee, I. Ha, S. Han, M. Choi, J. Lee, S. Hong, K.-J. Cho, S. H. Ko, *Nano Lett.* **2019**, *19*, 6087.
[24] M. Sun, P. Golvari, S. M. Kuebler, P. G. Kik, *ACS Photonics* **2023**, acsphotonics.2c01468.
[25] I. Schmidt, A. Gad, G. Scholz, H. Boht, M. Martens, M. Schilling, H. Suryo Wasisto, A. Waag, U. Schröder, *Biosens Bioelectron* **2017**, *94*, 74.
[26] S.-H. Wu, G. Cossio, B. Braun, F. C. M. Wu, E. T. Yu, *Advanced Optical Materials* **2023**, *11*, 2202409.
[27] S. Luo, E. Lian, J. He, J. C. deMello, *Adv Mater* **2023**, e2300058.
[28] Y. U. Cho, J. Y. Lee, U.-J. Jeong, S. H. Park, S. L. Lim, K. Y. Kim, J. W. Jang, J. H. Park, H. W. Kim, H. Shin, H. Jeon, Y. M. Jung, I.-J. Cho, K. J. Yu, *Advanced Functional Materials* **2022**, *32*, 2105568.
[29] P. D. Donaldson, Z. S. Navabi, R. E. Carter, S. M. L. Fausner, L. Ghanbari, T. J. Ebner, S. L. Swisher, S. B. Kodandaramaiah, *Advanced Healthcare Materials* **2022**, *11*, 2200626.
[30] E. Auroux, G. Huseynova, J. Ràfols-Ribé, V. M. L. Hera, L. Edman, *RSC Adv.* **2023**, *13*, 16943.
[31] S. Son, J. E. Park, J. Lee, M. Yang, B. Kang, *Sci Rep* **2016**, *6*, 34629.
[32] J. Park, D. Han, S. Choi, Y. Kim, J. Kwak, *RSC Adv.* **2019**, *9*, 5731.
[33] L. Martínez-Sarti, A. Pertegás, M. Monrabal-Capilla, E. Gilshteyn, I. Varjos, E. I. Kauppinen, A. G. Nasibulin, M. Sessolo, H. J. Bolink, *Organic Electronics* **2016**, *30*, 36.
[34] L. Yang, T. Zhang, H. Zhou, S. C. Price, B. J. Wiley, W. You, *ACS Appl. Mater. Interfaces* **2011**, *3*, 4075.
[35] C. Feng, K. Liu, J.-S. Wu, L. Liu, J.-S. Cheng, Y. Zhang, Y. Sun, Q. Li, S. Fan, K. Jiang, *Advanced Functional Materials* **2010**, *20*, 885.
[36] D.-W. Park, A. A. Schendel, S. Mikael, S. K. Brodnick, T. J. Richner, J. P. Ness, M. R. Hayat, F. Atry, S. T. Frye, R. Pashaie, S. Thongpang, Z. Ma, J. C. Williams, *Nat Commun* **2014**, *5*, 5258.
[37] D. Kuzum, H. Takano, E. Shim, J. C. Reed, H. Juul, A. G. Richardson, J. de Vries, H. Bink, M. A. Dichter, T. H. Lucas, D. A. Coulter, E. Cubukcu, B. Litt, *Nat Commun* **2014**, *5*, 5259.
[38] S. Chun, D. W. Kim, J. Kim, C. Pang, *Nanotechnology* **2019**, *30*, 335501.
[39] A. Cirocka, D. Zarzeczańska, A. Wcisło, *Materials* **2021**, *14*, 4743.
[40] Z.-H. Jin, Y.-L. Liu, J.-J. Chen, S.-L. Cai, J.-Q. Xu, W.-H. Huang, *Anal Chem* **2017**, *89*, 2032.
[41] Z. Lin, D. Kireev, N. Liu, S. Gupta, J. LaPiano, S. N. Obaid, Z. Chen, D. Akinwande, I. R. Efimov, *Advanced Materials* **2023**, *35*, 2212190.
[42] Y. Lu, X. Liu, R. Hattori, C. Ren, X. Zhang, T. Komiyama, D. Kuzum, *Advanced Functional Materials* **2018**, *28*, 1800002.
[43] K. Kim, S. Lee, J.-S. Nam, M. Joo, B. Mikladal, Q. Zhang, E. I. Kauppinen, I. Jeon, S. An, *Advanced Functional Materials* **2023**, *33*, 2213374.

[44] Y. Lee, S. Bae, H. Jang, S. Jang, S.-E. Zhu, S. H. Sim, Y. I. Song, B. H. Hong, J.-H. Ahn, *Nano Lett.* **2010**, *10*, 490.
[45] Y. Xu, Y. Wang, J. Liang, Y. Huang, Y. Ma, X. Wan, Y. Chen, *Nano Res.* **2009**, *2*, 343.
[46] E. Fortunato, D. Ginley, H. Hosono, D. C. Paine, *MRS Bulletin* **2007**, *32*, 242.
[47] C. G. Granqvist, A. Hultåker, *Thin Solid Films* **2002**, *411*, 1.
[48] K. Ellmer, *Nature Photonics* **2012**, *6*, 809.
[49] † Jung-Yong Lee, ‡ Stephen T. Connor, § and Yi Cui, † Peter Peumans*, "Solution-Processed Metal Nanowire Mesh Transparent Electrodes," DOI 10.1021/nl073296gcan be found under https://pubs.acs.org/doi/abs/10.1021/nl073296g, **2008**.
[50] S. De, J. N. Coleman, "Are There Fundamental Limitations on the Sheet Resistance and Transmittance of Thin Graphene Films?," DOI 10.1021/nn100343fcan be found under https://pubs.acs.org/doi/abs/10.1021/nn100343f, **2010**.
[51] K. R. Catchpole, S. Mokkapati, F. Beck, E.-C. Wang, A. McKinley, A. Basch, J. Lee, *MRS Bulletin* **2011**, *36*, 461.
[52] S. Kirchmeyer, K. Reuter, *J. Mater. Chem.* **2005**, *15*, 2077.
[53] D. S. Hecht, L. Hu, G. Irvin, *Advanced Materials* **2011**, *23*, 1482.
[54] R. Tenent, T. Barnes, J. Bergeson, A. Ferguson, B. To, L. Gedvilas, M. Heben, J. Blackburn, *Advanced Materials* **2009**, *21*, 3210.
[55] D. Li, R. B. Kaner, *Science* **2008**, *320*, 1170.
[56] Z. Wu, Z. Chen, X. Du, J. M. Logan, J. Sippel, M. Nikolou, K. Kamaras, J. R. Reynolds, D. B. Tanner, A. F. Hebard, A. G. Rinzler, *Science* **2004**, *305*, 1273.
[57] M. W. Rowell, M. A. Topinka, M. D. McGehee, H.-J. Prall, G. Dennler, N. S. Sariciftci, L. Hu, G. Gruner, *Applied Physics Letters* **2006**, *88*, 233506.
[58] X. He, Q. Cao, J. Pan, L. Yang, S. He, *RSC Adv.* **2021**, *11*, 11481.
[59] T. Sannicolo, M. Lagrange, A. Cabos, C. Celle, J.-P. Simonato, D. Bellet, *Small* **2016**, *12*, 6052.
[60] K. L. Chopra, P. D. Paulson, V. Dutta, *Progress in Photovoltaics: Research and Applications* **2004**, *12*, 69.
[61] S. De, T. M. Higgins, P. E. Lyons, E. M. Doherty, P. N. Nirmalraj, W. J. Blau, J. J. Boland, J. N. Coleman, *ACS Nano* **2009**, *3*, 1767.
[62] T. Sekitani, T. Someya, *Advanced Materials* **2010**, *22*, 2228.
[63] Y. Ma, G. W. Sim, S. Jo, D. C. Hyun, J.-S. Roh, D. Ko, J. Kim, *Applied Sciences* **2024**, *14*, 420.
[64] S. Park, M. Vosguerichian, Z. Bao, *Nanoscale* **2013**, *5*, 1727.
[65] A. K. Geim, K. S. Novoselov, *Nature Mater* **2007**, *6*, 183.
[66] Q. Li, M. R. Vogt, H. Wang, C. Monticelli, A. Zanelli, *Journal of Cleaner Production* **2024**, *468*, 143068.
[67] W. Macdonald, M. Looney, D. MacKerron, R. Eveson, R. Adam, K. Hashimoto, K. Rakos, *Journal of The Society for Information Display - J SOC INF DISP* **2007**, *15*, DOI 10.1889/1.2825093.
[68] M. Kim, H.-K. Um, H. Choi, J. S. Lee, J. Kim, K. Kim, E. Noh, M. Han, H. W. Lee, W. I. Choi, S. H. Lee, J.-R. Lee, B. H. Lee, *Advanced Electronic Materials* **2023**, *9*, 2300075.
[69] E. K. Lee, R. K. Baruah, H. Bhamra, Y.-J. Kim, H. Yoo, *Biomed. Eng. Lett.* **2021**, *11*, 107.
